# Potentiating Effect of UVA Irradiation on Anticancer Activity of *Carboplatin* Derivatives Involving 7-Azaindoles

**DOI:** 10.1371/journal.pone.0123595

**Published:** 2015-04-15

**Authors:** Pavel Štarha, Zdeněk Trávníček, Zdeněk Dvořák, Tereza Radošová-Muchová, Jitka Prachařová, Ján Vančo, Jana Kašpárková

**Affiliations:** 1 Regional Centre of Advanced Technologies and Materials & Department of Inorganic Chemistry, Faculty of Science, Palacký University, Olomouc, Czech Republic; 2 Regional Centre of Advanced Technologies and Materials & Department of Cell Biology and Genetics, Faculty of Science, Palacký University, Olomouc, Czech Republic; 3 Centre of the Region Haná for Biotechnological and Agricultural Research & Department of Biophysics, Faculty of Science, Palacký University, Olomouc, Czech Republic; 4 Department of Biophysics, Faculty of Science, Palacký University, Olomouc, Czech Republic; National Institute of technology Rourkela, INDIA

## Abstract

The moderate-to-high *in vitro* cytotoxicity against ovarian A2780 (IC_50_ = 4.7–14.4 μM), prostate LNCaP (IC_50_ = 18.7–30.8 μM) and prostate PC-3 (IC_50_ = 17.6–42.3 μM) human cancer cell lines of the platinum(II) cyclobutane-1,1'-dicarboxylato complexes [Pt(cbdc)(*n*aza)2] (**1–6**; cbdc = cyclobutane-1,1'-dicarboxylate(2-); *n*aza = halogeno-substituted 7-azaindoles), derived from the anticancer metallodrug *carboplatin*, are reported. The complexes containing the chloro- and bromo-substituted 7-azaindoles (**1**, **2**, and **4–6**) showed a significantly higher (p < 0.05) cytotoxicity against A2780 cell line as compared to *cisplatin* used as a reference drug. Addition of the non-toxic concentration (5.0 μM) of L-buthionine sulfoximine (L-BSO, an effective inhibitor of γ-glutamylcysteine synthase) markedly increases the *in vitro* cytotoxicity of the selected complex **3** against A2780 cancer cell line by a factor of about 4.4. The cytotoxicity against A2780 and LNCaP cells, as well as the DNA platination, were effectively enhanced by UVA light irradiation (λ_max_ = 365 nm) of the complexes, with the highest phototoxicity determined for compound **3**, resulting in a 4-fold decline in the A2780 cells viability from 25.1% to 6.1%. The ^1^H NMR and ESI-MS experiments suggested that the complexes did not interact with glutathione as well as their ability to interact with guanosine monophosphate. The studies also confirmed UVA light induced the formation of the *cis* [Pt(H_2_O)_2_(cbdc`)(*n*aza)] intermediate, where cbdc` represents monodentate-coordinated cbdc ligand, which is thought to be responsible for the enhanced cytotoxicity. This is further supported by the results of transcription mapping experiments showing that the studied complexes preferentially form the bifunctional adducts with DNA under UVA irradiation, in contrast to the formation of the less effective monofunctional adducts in dark.

## Introduction

Although the well-known story of platinum-based anticancer metallotherapeutics have slowly reached their second half-century, the application, development and research is still one of the leading branches of bioinorganic chemistry [1–3]. However, there is still room for improvement with regard to the therapeutic effects of platinum-based antitumor active complexes, combined with the suppression of negative side-effects (*e*.*g*. nephrotoxicity, neurotoxicity or myelosuppression) and/or ability to overcome both the intrinsic or acquired resistance of various tumor cells against chemotherapeutics [2–5]. One of the current approaches for reaching the aforementioned objectives is based on the irradiation at selected wavelengths of light and converting the initially inactive drugs into significantly enhanced cytotoxics [6–8]. Recently, the considerably increased ability of the 2^nd^ generation platinum-based anticancer drug *carboplatin* to bind to the DNA upon UVA irradiation, resulting in increased cytotoxicity, was reported [8].

**Fig 1:**
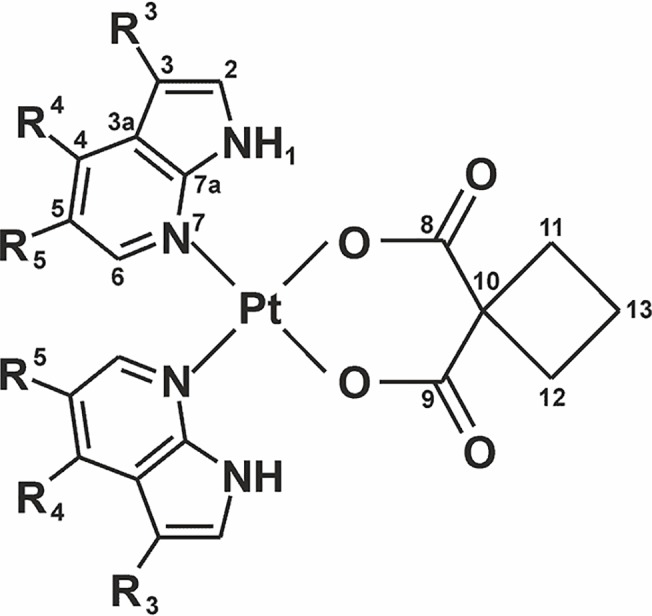
Structural formula of the studied complexes [Pt(cbdc)(*n*aza)_2_] (1–6). The formula is given together with their atom numbering scheme; R_3_, R_4_, R_5_ = Cl, H, H (3-chloro-7-azaindole (*3Cl*aza), involved in complex **1**); Br, H, H (3-bromo-7-azaindole (*3Br*aza), **2**); I, H, H (3-iodo-7-azaidole (*3I*aza), **3**); H, Cl, H (4-chloro-7-azaindole (*4Cl*aza), **4**); H, Br, H (4-bromo-7-azaindole (*4Br*aza), **5**); and H, H, Br (5-bromo-7-azaindole (*5Br*aza), **6**).

*Carboplatin* represents a complex, whose composition offers the possibility of facile derivatization. The approach is based on the derivatization of the cyclobutane-1,1'-dicarboxylate(2-) (cbdc) (*e*.*g*. the diammineplatinum(II) complexes with the furoxan-substituted cyclobutane moiety [9]), while the second involves the replacement of the NH_3_ carrier-ligands (*e*.*g*. with adenine-based *N*-donor ligands [10]). In this work, the second approach was applied to yield a series of cyclobutane-1,1'-dicarboxylatoplatinum(II) complexes where both the NH_3_ ligands are substituted by various 7-azaindoles (*n*aza). 7-Azaindole was recently used as a suitable *N*-donor carrier ligand of various types of antitumor active platinum(II) complexes, and a number of dichlorido [11–13], mixed-ligand [14,15] and oxalato [11] complexes have been reported to date. The herein presented complexes represent a logical step towards the extension of the group of dichlorido and oxalato platinum(II) complexes, involving the analogical halogeno-derivatives of 7-azaindole, recently developed by our research group [11–13]. In the case of dichlorido complexes, considerably high *in vitro* cytotoxicity (with IC_50_ values up to 0.6 μM) was found against various human cancer cell lines (ovarian A2780, breast MCF7, osteosarcoma HOS, lung A549, cervical HeLa, malignant melanoma G361 and prostate LNCaP). These *cisplatin* analogues complexes also successfully overcame an acquired resistance to cancer cells (ovarian carcinoma model) and effectively reduced the tumor tissues volume during the *in vivo* experiments on mice (L1210 lymphocytic leukemia model), while showing less serious negative side-effects on the healthy tissues as compared with *cisplatin* [13]. The above-mentioned positive findings, regarding the *in vitro* and *in vivo* anticancer activities of platinum(II) complexes bearing 7-azaindole monodentate ligands, motivated us to study the *carboplatin* analogues involving the mentioned *N*-donor ligands (Fig 1), their cytotoxicity on selected human cancer cell lines and mechanisms of their action under normal conditions and upon UVA light irradiation, using the set of advanced analytical and biological methods.

## Materials and Methods

### Chemicals and Biochemicals

The reagents (K_2_[PtCl_4_], *3Cl*aza, *3Br*aza, *3I*aza, *4Cl*aza, *4Br*aza and *5Br*aza, cyclobutane-1,1'-dicarboxylic acid, NaOH, AgNO_3_), solvents (*N*,*N’*-dimethylformamide (DMF), acetone, methanol, diethyl ether) and other chemical (reduced glutathione (GSH), guanosine 5'-monophosphate disodium salt hydrate (GMP)) were supplied by Sigma-Aldrich (Prague, Czech Republic) and Acros Organics (Pardubice, Czech Republic). Calf thymus DNA (CT DNA; 42% G:C, mean molecular mass of approximately 20,000 kDa) was isolated as previously described [16]. Sephadex G-50 (coarse) was from Sigma-Aldrich (Prague, Czech Republic). MTT, 3-(4,5-Dimethylthiazol-2-yl)-2,5-diphenyltetrazolium bromide, was from Calbiochem (Darmstadt, Germany). RPMI 1640 medium, fetal bovine serum, trypsin/EDTA, and Dulbecco’s modified Eagle’s medium were from PAA (Pasching, Austria). Gentamicin was from Serva (Heidelberg, Germany).

### General Method for the Synthesis of 1–6

The platinum(II) cyclobutane-1,1'-dicarboxylato complexes, [Pt(cbdc)(*n*aza)_2_] (**1**–**6**; *n*aza = *3Cl*aza for **1**, *3Br*aza for **2**, *3I*aza for **3**, *4Cl*aza for **4**, *4Br*aza for **5** and *5Br*aza for **6**), were prepared by well-established Dhara`s method [17]. Briefly, 1.0 mmol (415 mg) of K_2_[PtCl_4_] was dissolved in 15 mL of deionized water at room temperature and KI (830 mg; 5.0 mmol) was added. The solution turned black during 1 h of stirring at room temperature and then 2.0 mmol of *n*aza dissolved in 15 mL of methanol were poured in. The mixture was stirred overnight at room temperature and the obtained yellow solid, *i*.*e*. *cis*-[PtI_2_(*n*aza)_2_] (yields ≈90%), was filtered off and washed with deionized water (3 × 5 mL) and methanol (3 × 5 mL), dried and stored in desiccator over silica gel. The obtained platinum(II) diiodido complexes (0.5 mmol) were dissolved in DMF (5 mL) and silver(I) cyclobutane-1,1'-dicarboxylate (0.5 mmol) was added into the solution. The mixtures were stirred at room temperature and in the dark for 48 h. The formed AgI precipitate was collected and washed with DMF (2 × 5 mL). Deionized water (25 mL) was poured into the filtrate and the obtained white precipitate was removed by filtration and washed successively with deionized water (2 × 5 mL), methanol (2 × 5 mL) and diethyl ether (2 × 5 mL). The products of [Pt(cbdc)(*n*aza)_2_] (**1**–**6**; Fig 1) were dried in desiccator over silica gel and stored without any further purification. Characterization data for **1**–**6** are given in Supporting Information (S1 Text).

### Physical Measurements

A combustion analysis (C, H, N) was performed using a Flash 2000 CHNS Elemental Analyzer (Thermo Scientific). Electrospray ionization mass spectroscopy (ESI-MS) of the methanol solutions was performed on an LCQ Fleet Ion Trap mass spectrometer (Thermo Scientific; QualBrowser software, version 2.0.7) in both the positive (ESI+) and negative (ESI–) ionization modes. Infrared spectra were recorded on a Nexus 670 FT-IR (Thermo Nicolet) using the ATR technique in the 400–4000 cm^–1^ region. The NMR spectra (^1^H, ^13^C, ^1^H–^1^H gs-COSY, ^1^H–^13^C gs-HMQC and ^1^H–^13^C gs-HMBC; gs = gradient selected, COSY = correlation spectroscopy, HMQC = heteronuclear multiple quantum coherence, HMBC = heteronuclear multiple bond coherence) were acquired on the DMF-*d*_*7*_ solutions at 300 K on either a JEOL JNM-ECA600II spectrometer at 600.00 MHz (^1^H) and 150.86 MHz (^13^C) (complexes **1**, **4** and **6**) or a Varian 400 spectrometer at 400.00 MHz (^1^H) and 100.58 MHz (^13^C) (complexes **2**, **3** and **5**). Proton and carbon spectra were calibrated against the residual DMF-*d*_*6*_^1^H NMR (8.03, 2.92 and 2.75 ppm) and ^13^C NMR (163.15, 34.89 and 29.76 ppm) signals. The symbols s (singlet), d (doublet), t (triplet), qui (quintet), br (broad signal) and m (multiplet) is used for the splitting of the proton resonances.

#### Solution Stability Studies

Stability of the studied complexes in DMF-*d*_*7*_ (**1**–**6**) and DMF-*d*_*7*_/H_2_O mixture (1:1, v/v; **5**) was monitored by ^1^H NMR spectroscopy (Varian 400 MHz device) after 24 h (both solutions) and 14 days (only DMF-*d*_*7*_ solutions) of standing at room temperature under ambient light.

### Methods of Biological Testing

#### Cell Culture and In Vitro Cytotoxicity Testing

*In vitro* cytotoxicity of the complexes **1**–**6,** *cisplatin* and *carboplatin* was tested by an MTT assay against ovarian carcinoma A2780 (ECACC No. 93112519), prostate carcinoma LNCaP (ECACC No. 89110211) and prostate carcinoma PC-3 (ECACC No. 90112714) human cancer cells obtained from European Collection of Cell Cultures (ECACC), as described in our previous works [11,13]. The cell lines were maintained in a humidified incubator (37°C, 5% CO_2_). The cells were treated with **1**–**6,** *cisplatin* and *carboplatin* at the 0.01–50.0 μM concentrations using 96-well culture plates. The cells were treated in parallel with vehicle (DMF; 0.1%, v/v), and Triton X-100 (1%, v/v) to assess the minimal (100% of cell viability), and maximal (0% of cell viability) cell damage, respectively. The exposure time was 24 h. The MTT assay was used to determine the cell viability by the spectrophotometric measurements of the solubilized dye at 540 nm (TECAN, Schoeller Instruments LLC).

Analogical *in vitro* cytotoxicity experiments were performed in the case of complex **3** on the A2780 cells with the addition of L-buthionine sulfoximine (L-BSO), which was independently added to each well to give the 5.0 μM final concentration of L-BSO (5.0 μM concentration of L-BSO is known to be non-toxic and optimal for the experiments focusing on the modulation of anticancer active transition metal complexes) [18]. These experiments were performed with two negative controls (DMF and 5.0 μM L-BSO), and no statistically different results were obtained between the controls.

The IC_50_ values (compound concentrations that produce 50% of cell growth inhibition; μM±SD) were acquired by three independent experiments (each conducted in triplicate) performed on the cells from different passages. The statistical evaluation (p < 0.05 were considered as significant) of the obtained data was carried out by ANOVA using QC Expert 3.2 statistical software (TriloByte Ltd.).

#### UVA Light Irradiation

A LZC-4V photoreactor (Luzchem, Ottawa, ON, Canada) employed with a temperature controller was used for irradiation (4.3 mW cm^-2^; λ_max_ = 365 nm) of the DNA samples in cell-free media and using the UVA tubes, as described previously [8].

#### Platination of DNA in Cell-free Media

CT DNA (0.2 mg mL^–1^) was mixed with **1**–**6** or, for comparative purposes, with *carboplatin* in NaClO_4_ (10 mM) and immediately irradiated (UVA, λ_max_ = 365 nm) for 3 h at 37°C in the dark and then kept for additional 2 h under the mentioned conditions, similarly as described in [8]. The r_i_ value was 0.08 (r_i_ = the molar ratio of free platinum complex to nucleotide phosphates at the onset of incubation with DNA). After the incubation, the samples were quickly filtered using a Sephadex G-50 column to remove free (unbound) platinum. The platinum content in these DNA samples (r_b_, defined as the number of the molecules of platinum complex coordinated per nucleotide residue) was determined by flameless atomic absorption spectrometry (FAAS).

#### *In Vitro* Phototoxicity

A2780 and LNCaP cells were seeded in 96-well tissue culture plates in 100 μL medium in the absence of antibiotics at a density of 5,000 cells per well and placed in the incubator for 24 h, as previously reported [8]. The solutions of **2**, **3**, **4** or **5** in the medium (100 μL) were added. The cells were incubated for 24 h. After that the cells were washed out, and the medium containing the platinum(II) complex was replaced by a drug-free medium in the absence of antibiotics, followed by 20 min irradiation with UVA or sham irradiation. After additional 24 h, cell viability was evaluated by an MTT (*vide supra*).

#### Stability and Interaction Studies after UVA Light Irradiation

The ^1^H NMR spectra (Varian 400 MHz device) of the selected representative complex **5**, its mixture with two molar equivalents of GSH (**5**+GSH), and its mixture with two molar equivalents of guanosine monophosphate (**5**+GMP) in DMF-*d*_*7*_/H_2_O mixture (1:1, v/v) were recorded right after the UVA irradiation (20 min) after 24 h of standing at room temperature under ambient light. In the case of irradiated **5** itself (*i*.*e*. without GSH or GMP) the ^1^H NMR spectrum was also recorded after 96 h of standing at room temperature under ambient light. The ESI mass spectra in both the positive and negative ionization modes were recorded using all the mentioned solutions (*i*.*e*. **5**, **5**+GSH and **5**+GMP) 24 h after UVA irradiation by the ThermoFinnigan LCQ Fleet Ion Trap mass spectrometer (Thermo Scientific).

#### Transcription Mapping of DNA Adducts *In Vitro*

Linear pSP73KB/HpaI DNA was incubated with the selected platinum complexes **3** or **5** so that the DNA samples with the platinum(II) complex were irradiated with UVA for 30 min with subsequent incubation for additional 4.5 h in the dark at 37°C, or, alternatively, the DNA samples were incubated with the platinum(II) complexes in the dark at 37°C for 5 h. After incubation, the samples were precipitated with ethanol to remove unbound complex and the obtained solid (pellet) was dissolved in 0.01 M NaClO_4_. The aliquots of the samples were used to determine level of Pt bound to DNA (r_b_, defined as the number of molecules of the platinum(II) complex bound per nucleotide residue) by using FAAS and spectrophotometric determination of DNA at 260 nm.

Transcription of the linear pSP73KB/HpaI DNA treated with the complexes with DNA-dependent T7 RNA polymerase, followed by the electrophoretic analysis of transcripts were carried out according to the manufacturer (Promega Protocols and Applications, 43–46, 1989/90) recommended protocols, as described previously [19]. The DNA concentration used in this assay (relative to the monomeric nucleotide content) was 39 μM.

#### Interstrand DNA Cross-linking in a Cell-free Medium

Linear pSP73KB DNA/EcoRI (2455 bp) was mixed with **3** or **5** and immediately irradiated with UVA for 30 min with subsequent incubation for additional 4.5 h in the dark at 37°C [8]. Alternatively, the DNA was incubated with the platinum(II) complexes in the dark at 37°C for 5 h. After incubation, the samples were precipitated to remove free, unbound platinum complex, dissolved in 0.01 M NaClO_4_ and the r_b_ in the aliquots of these samples was estimated by FAAS and spectrophotometric determination of DNA at 260 nm. DNA in the remaining part of samples was 3´-end-labeled by means of the Klenow fragment of DNA polymerase I in the presence of [α-^32^P]dATP. The labeled samples were evaluated for DNA interstrand cross-links according to the previously published procedures by electrophoresis under denaturing conditions on alkaline agarose gel (1%) [19,20]. After the electrophoresis had been completed, the intensities of the bands corresponding to single strands of DNA and interstrand cross-linked duplex were quantified. The frequency of interstrand cross-links was calculated as ICL/Pt (%) = XL/4918 r_b_ (the DNA fragment contained 4918 nucleotide residues), where ICL/Pt (%) is the number of interstrand cross-links per adduct multiplied by 100, and XL is the number of interstrand cross-links per molecule of the linearized DNA duplex, and was calculated assuming a Poisson distribution of the interstrand cross-links as XL = -ln A, where A is the fraction of molecules running as a band corresponding to the non-cross-linked DNA.

#### Fluorescence Quenching Experiments

Fluorescence measurements of systems consisting of ethidium bromide (EtBr) and CT DNA with addition of platinum complexes **3** or **5** were carried out at a 546 nm excitation wavelength, and the emitted fluorescence was analyzed at 590 nm. These measurements were performed on a Varian Cary fluorescence spectrophotometer using a 1 cm quartz cell. The fluorescence intensity was measured at 25°C in 0.4 M NaCl to avoid secondary binding of EtBr to DNA [21,22]. The concentrations were 0.01 mg mL^−1^ for DNA and 0.04 mg mL^−1^ for EtBr, which corresponded to the saturation of all sites of EtBr in DNA [21].

## Results

### Chemistry

A series of six complexes of the general formula [Pt(cbdc)(*n*aza)_2_] (**1**–**6**; Fig 1) was prepared in *ca*. 40% yields (relating to K_2_[PtCl_4_]) and their chemical purity (>95%) was checked by combustion analysis (see S1 Text) and by ^1^H NMR spectroscopy (S1 Fig).

**Fig 2:**
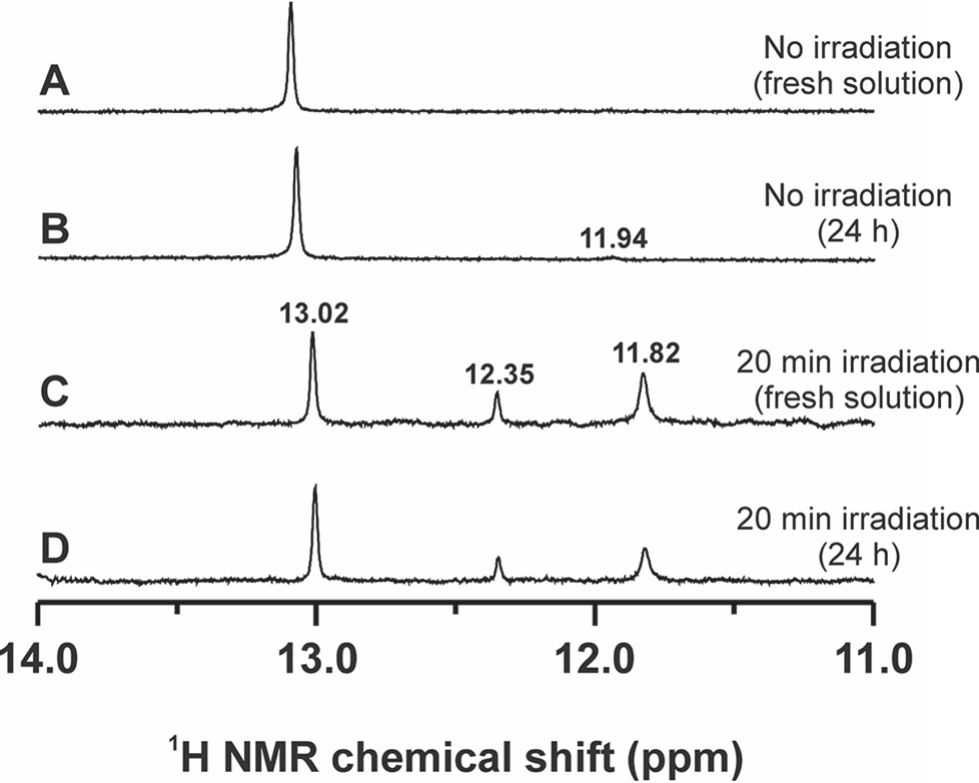
^1^H NMR stability studies. Time-dependent (**A** and **C**—fresh solutions; **B** and **D**—after 24 h of standing at room temperature under ambient light) 400 MHz ^1^H NMR spectra (N1–H region of *4Br*aza) as observed before (**A** and **B**) and after (**C** and **D**) UVA irradiation (20 min, λ_max_ = 365 nm) of complex **5** dissolved in the DMF-*d7*/H_2_O solution (1:1, v/v).

All the corresponding ^1^H and ^13^C signals (with the appropriate integral intensities) of coordinated *n*aza and cbdc ligands were detected in the spectra (S1 Fig). An N7 coordination mode of the *n*aza ligands was clearly proved for the complexes **1**–**6** from the calculated ^1^H and ^13^C NMR coordination shifts (S1 Table). All the complexes were found to be stable in DMF-*d*_*7*_ over 14 days (no changes were detected in the ^1^H NMR spectra). In the case of the selected complex **5** dissolved in the DMF-*d*_*7*_/H_2_O mixture (1:1, v/v), a new set of signals corresponding to *4Br*aza ligand (*e*.*g*. N1–H signal at 11.94 ppm or C6–H signal 8.19 ppm) was detected after 24 h of standing at room temperature under ambient light (Fig 2B). The chemical shifts detected were different from those of free *4Br*aza molecule in the same solvent (*e*.*g*. N1–H signal at 11.82 ppm).

The ESI-MS spectra, measured in both the positive (ESI+) and negative (ESI–) ionization mode, of all the studied complexes contained the molecular peaks, *i*.*e*. {[Pt(cbdc)(*n*aza)_2_]+H}^+^, and {[Pt(cbdc)(*n*aza)_2_]–H}^–^, respectively (S3 Fig). Moreover, the adducts with sodium ions, {[Pt(cbdc)(*n*aza)_2_]+Na}^+^, as well as the {[Pt(cbdc)(*n*aza)]–H}^—^and {*n*aza+H}^+^ species were identified in the appropriate spectra as well.

### Biological activities testing

#### *In Vitro* Cytotoxicity

The *in vitro* cytotoxicity of the prepared complexes **1**–**6** (applied within the concentration range of 0.01–50.0 μM, depending on the solubility in water-containing medium) can be generalized as high against the A2780 ovarian carcinoma cancer cell line (IC_50_ = 4.7–14.4 μM) and moderate in the case of LNCaP (IC_50_ = 18.7–30.8 μM) and PC-3 (IC_50_ = 17.6–42.3 μM) prostate carcinoma cancer cell lines (Table 1). All the complexes exceeded the activity of the reference drug *cisplatin* against A2780, with the most effective complex **6** being *ca*. 4.6-fold more cytotoxic. Except for compound **3**, the *in vitro* cytotoxicity of the studied complexes against the A2780 cells was significantly higher (ANOVA, p < 0.05) as compared to *cisplatin*. In the case of LNCaP and PC-3, the evaluation was limited by the fact that IC_50_ of *cisplatin* could not be obtained, because it is higher than the highest applied concentration, above which the compounds are generally considered to be ineffective (*i*.*e*. IC_50_ > 50.0 μM). Complex **4** was found to be the most cytotoxic on LNCaP and PC-3 cell lines (Table 1). The studied complexes also exceeded the *in vitro* cytotoxicity of *carboplatin*, which was found to be non-toxic up to the 50.0 μM concentration against all three cancer cell lines used (Table 1).

**Table 1:** *In vitro* antitumor activity of 1–6, *cisplatin* and *carboplatin* against A2780, LNCaP and PC-3 cancer cell lines.

Complex	A2780	LNCaP	PC-3
**1**	11.8±6.2*	30.8±3.6	36.3±2.3
**2**	10.3±4.3*	>20.0^a^	18.5±0.9
**3**	14.4±6.0	>50.0^a^	42.3±0.8
**4**	5.3±0.9*	18.7±5.1	17.6±8.8
**5**	5.1±0.9*	23.5±3.8	26.6±4.1
**6**	4.7±1.9*	22.1±1.4	29.6±9.4
*Carboplatin*	>50.0^a^	>50.0^a^	>50.0^a^
*Cisplatin*	21.8±3.9	>50.0^a^	>50.0^a^

The results of the *in vitro* antitumor activity testing of **1**–**6**, *cisplatin* and *carboplatin* against human ovarian (A2780) and prostate (LNCaP and PC-3) cancer cell lines. Cells were treated with the tested compounds for 24 h, measurements were performed in triplicate, and cytotoxicity experiment was repeated in three different cell passages. Data are expressed as IC_50_ ± SD (μM).

asterisk (*), significantly different values (p < 0.05) between **1**–**6** and *cisplatin*

^a)^ IC_50_ were not reached up to the given concentration

The *in vitro* cytotoxicity of the selected representative complex **3** was found to be significantly higher in the presence of 5.0 μM L-BSO, an effective inhibitor of glutathione synthesis, as the IC_50_ value determined for the A2780 cell line equalled to 3.3±0.3 μM. This means about 4.4-times enhancement in the antiproliferative activity as compared to the same complex without added L-BSO.

#### Phototoxicity in Cell Cultures

**Fig 3:**
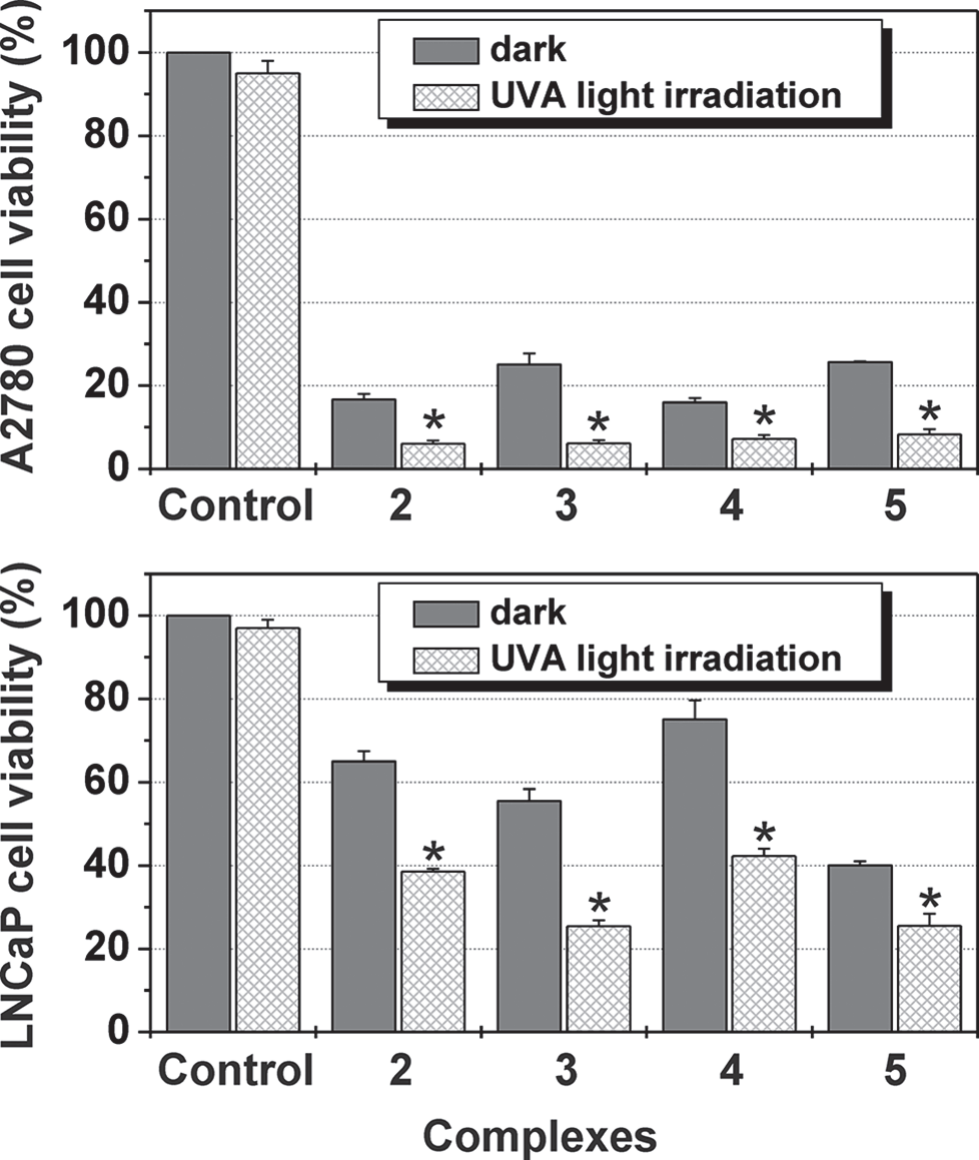
Cytotoxic activity of the complexes 2–5. Cytotoxic activity of **2**–**5** at their 10 μM concentrations against the A2780 (top panel) and LNCaP (bottom panel) cell lines. Viability of the untreated, sham-irradiated cells was taken as 100%. The asterisk (*) denotes significant difference (p < 0.05) between the irradiated and sham—irradiated cells.

The effect on the cell viability determined for each tested compound was significantly (p < 0.05) higher when the complex was applied in combination with UVA irradiation, as compared to the reference sample (in the dark) (Fig 3). Importantly, the control cells (with and without UVA exposure) grew at the same rate.

#### DNA Binding in Cell-free Media

Samples of double-helical CT DNA were incubated with the complex at r_i_ value of 0.08 in 0.01 M NaClO_4_ at 37°C and subsequently divided into two parts. One part was irradiated with UVA light (λ_max_ = 365 nm, 4.3 mW cm^-2^) immediately after addition of the complex; the other (control) sample was kept in the dark. After 5 h of incubation, the samples were assayed for platinum content bound to DNA, as described above, by FAAS. The amount of platinum bound to DNA in the samples, which were kept in the dark, ranged from 22 to 42% (Table 2). In contrast, after 5 h of continuous UVA irradiation, the platination of DNA increased *ca*. 2–3-fold, as compared to the samples incubated in the dark (Table 2).

**Table 2:** DNA binding of 1–6 in cell free media.

Complex	dark	UVA
**1**	36±3	72±11
**2**	22±3	82±11
**3**	37±7	80±12
**4**	35±6	86±1
**5**	31±2	93±4
**6**	42±8	78±3
*Carboplatin*	4±1	54±4

Data are expressed as percentage of platinum bound to DNA to total platinum income. Data represent the mean ± SD of three independent experiments.

#### Stability Studies after the UVA Irradiation

**Fig 4:**
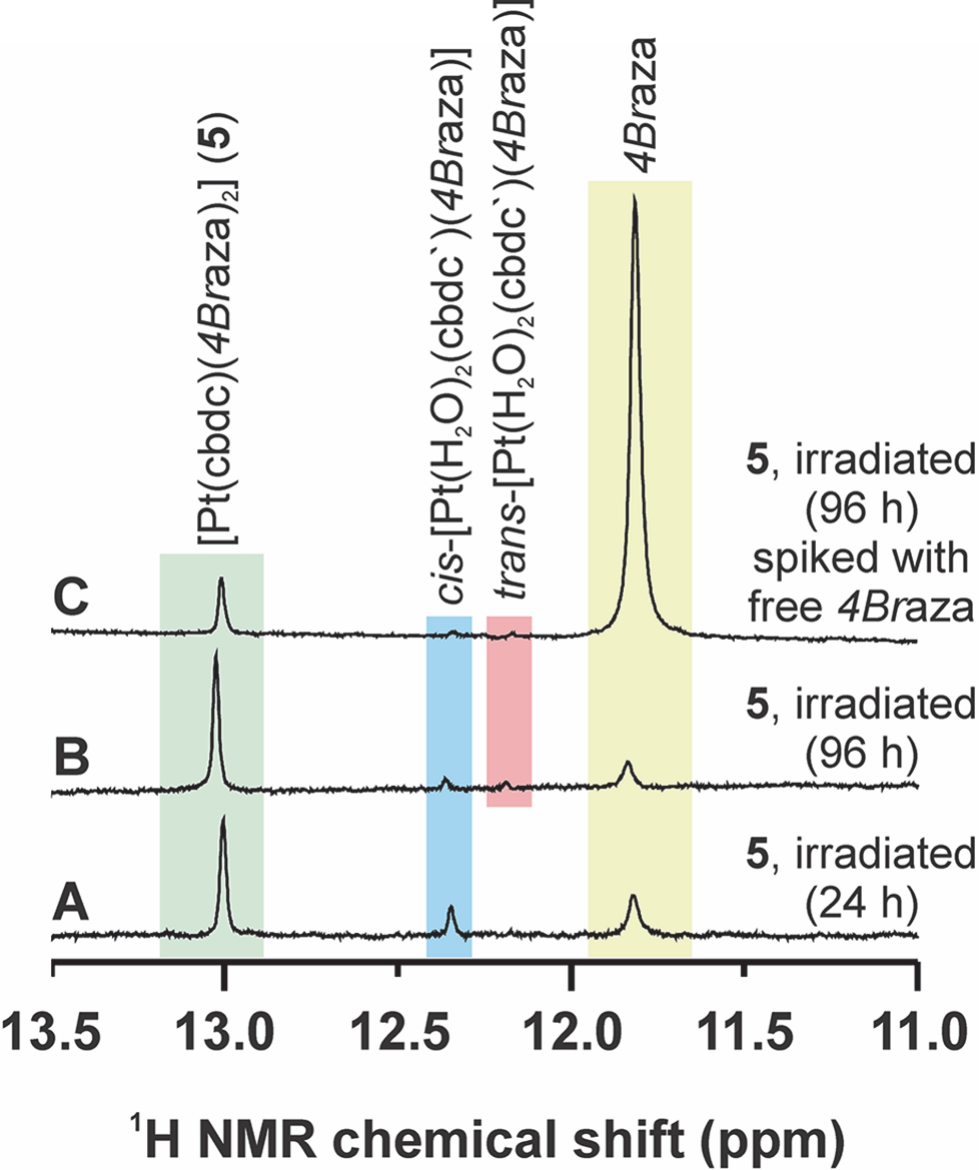
UVA irradiation effect on the composition of the complex 5. Time-dependent (after 24 (**A**) or 96 h (**B** and **C**) of standing at room temperature under ambient light) 400 MHz ^1^H NMR spectra (N1–H region of *4Br*aza) as observed in the DMF-*d7*/H_2_O (1:1, v/v) solutions of complex **5** (**A** and **B**), and complex **5** spiked with free *4Br*aza (**C**), with UVA irradiation (20 min, λ_max_ = 365 nm), respectively.

The ^1^H NMR spectrum of the complex [Pt(cbdc)(*4Br*aza)_2_] (**5**) dissolved in DMF-*d*_*7*_/H_2_O (1:1, v/v) was recorded before and after 20 min of UVA irradiation (λ_max_ = 365 nm; 4.3 mW cm^-2^). Before the irradiation, **5** showed one signal at 13.02 ppm, corresponding to the N1–H atom of the coordinated *4Br*aza ligand, while two new N1–H signals were detected at 11.82 and 12.35 ppm after the irradiation (Figs 2 and 4). The ^1^H NMR spectrum of the starting complex **5** (before the irradiation) showed, as assumed, one quintet (C13–H_2_) and one triplet (C11–H_2_, C12–H_2_) at 1.89, and 2.49 ppm, respectively (S4 Fig). One new C13–H_2_ quintet (2.08 ppm) was detected in the ^1^H NMR spectrum of the irradiated sample. The ^1^H NMR spectroscopy performed on the irradiated sample did not show any new signals nor any change in the integral intensities, as compared with the fresh irradiated solution, after 24 h (Fig 2), but at 96 h lower intensity of the signal at 12.35 ppm and one new signal at 12.18 ppm were detected. Additional ^1^H NMR experiments were performed on the irradiated solution of **5** (96 h after the irradiation) spiked with free *4Br*aza dissolved in the same solution (DMF-*d*_*7*_/H_2_O, 1:1, v/v), leading to marked intensity increase of the signal at 11.82 ppm, which suggested that the signal at 11.82 ppm belongs to the *4Br*aza molecule released from the studied complex (Fig 4).

The ratio of the integral intensities, showing on the portion of the rearranged/decomposed complex, of the N1–H signal of the parent compound and both the newly emerged signals is 1.00 (13.02 ppm) to 0.65 (12.35 ppm) to 1.06 (11.82 ppm), which indicates that the amount of the decomposed complex is *ca* 63% after 20 min UVA irradiation.

The ESI+ and ESI—mass spectra of complex **5** before the irradiation contained the {[Pt(cbdc)(*4Br*aza)_2_]+H}^+^ (733.2 *m/z*), {[Pt(cbdc)(*4Br*aza)_2_]–H}^–^(731.2 *m/z*) and {[Pt(cbdc)(*4Br*aza)_2_]+Na}^+^ (755.2 *m/z*) peaks of the starting complex or their adducts with the Na^+^ ion, as well as the peaks of the {[Pt(cbdc)(*4Br*aza)]–H}^–^(533.6 *m/z*) and {*4Br*aza+H}^+^ (197.1 *m/z*) species (S5 Fig). All these peaks were detected also in the spectra of the irradiated sample, but with significant changes in intensities. Concretely, the peak of the {*4Br*aza+H}^+^ fragment showed about 5-fold higher relative abundance in the ESI+ mass spectrum after the irradiation, which corresponded with markedly higher intensity of the peak of the {[Pt(cbdc)(*4Br*aza)]–H}^—^species with one *4Br*aza molecule released (S5 Fig).

#### Photoreaction with Biomolecules (GSH and GMP)

Analogous ^1^H NMR and ESI-MS experiments, as described above for complex **5**, were performed also for the mixtures of **5** with GSH (symbolized as **5**+GSH) or GMP (**5**+GMP) in DMF-*d*_*7*_/H_2_O (1:1, v/v).

The ^1^H NMR spectra of **5**+GSH, both before and after the irradiation, contained the characteristic GSH signals, such as triplet at 8.68 ppm and doublet at 8.56 ppm of the N–H hydrogen atoms of glycine, and cysteine part of the GSH molecule, respectively. As for *4Br*aza, the N1–H region of the ^1^H NMR spectrum (before irradiation) does not contain any new signals, as discussed above. As for the ^1^H NMR spectrum of the irradiated **5**+GSH mixture, only the same two new signals (*i*.*e*. at 12.35 and 11.82 ppm) in the N1–H region of *4Br*aza as in the case of the irradiated starting material itself, and no new signals in the region of the N–H hydrogen atoms of glycine and cysteine were observed. Furthermore, any peaks whose mass corresponded to the adducts of GSH and **5** or fragments were detected in the ESI+ and ESI—spectra of **5**+GSH, both before and after irradiation (S5 Fig).

Regarding the **5**+GMP mixture, the signals of the coordinated *4Br*aza as well as C8–H signal of free GMP (at 8.37 ppm) were clearly detected in the ^1^H NMR spectra before the irradiation. The UVA irradiation caused several changes: the signals (*e*.*g*. 8.19 ppm for C6–H; N1–H signals were not detected) of the released *4Br*aza ligand were detected and one new signal was observed very close to the C8–H signal of free GMP (at 8.31 ppm) with the integral intensity about three times lower compared to the C6–H signal of *4Br*aza of the starting material in the mixture (S6 Fig). The mass spectra of the mixtures containing the GMP also showed the new peaks (in addition to those discussed above, corresponding to the {*4Br*aza+H}^+^, {[Pt(cbdc)(*4Br*aza)_2_]+H}^+^, {[Pt(cbdc)(*4Br*aza)_2_]+Na}^+^, {[Pt(cbdc)(*4Br*aza)_2_]–H}^–^, {[Pt(cbdc)(*4Br*aza)]–H}^—^species) of the {GMP–Na+2H}^+^, {GMP+H}^+^, {GMP–2Na+H}^—^and also those corresponding to the {[Pt(cbdc)(*4Br*aza)(GMP)]–Na+2H}^+^, {[Pt(cbdc)(*4Br*aza)(GMP)]+H}^+^, and {[Pt(cbdc)(*4Br*aza)(GMP)]–2Na+H}^—^species at 368.3, 408.3, 362.4, 920.2, 942.3, and 896.3 *m/z*, respectively (S5 and S6 Figs).

#### Characterizations of DNA Adducts Formed in Dark and under the UVA Irradiation

Besides the DNA binding capacity, the important factor which modulates the cytotoxicity of platinum compounds is the nature of the conformational changes induced in DNA. In order to determine the nature of DNA adducts formed by [Pt(cbdc)(*n*aza)_2_] complexes in the dark and under the UVA irradiation, several biochemical and biophysical methods have been applied. As model compounds, complexes **3** and **5** have been selected since they exhibited the highest phototoxic effects.

#### Transcription Mapping of Platinum–DNA Adducts

**Fig 5:**
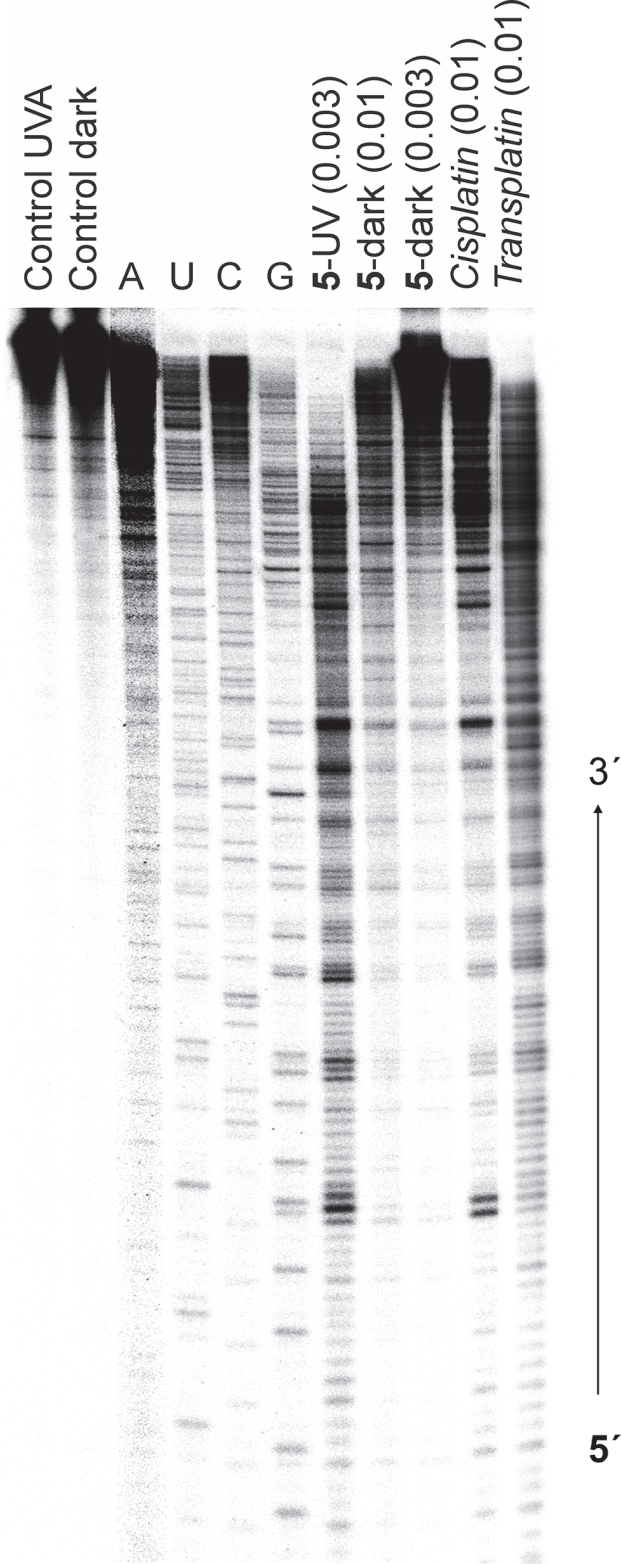
Inhibition of RNA synthesis by 5, *cisplatin* and *transplatin*. Inhibition of RNA synthesis by T7 RNA polymerase on the pSP73KB/HpaI fragment modified by **5** under the irradiation or in the dark, *cisplatin* or *transplatin*. Autoradiogram of 6% polyacrylamide/8 M urea gel. Lanes: Control UVA, unmodified template irradiated with UVA; Control dark, non-irradated unmodified template; A, U, G, C, chain terminated marker RNAs; **5-**UV (0.003), the template modified at r_b_ = 0.003 by irradiated **5**; **5-**dark (0.01), the template modified at r_b_ = 0.01 by **5** in the dark; **5-**dark (0.003), the template modified by **5** at r_b_ = 0.003 in the dark; *cisplatin* (0.01), the template modified at r_b_ = 0.01 by *cisplatin*; *transplatin* (0.01), the template modified at r_b_ = 0.01 by *transplatin*.

Experiments on *in vitro* RNA synthesis by T7 RNA polymerase were carried out using a linear pSP73KB/HpaI DNA fragment, treated with complex **3** or **5** in dark or under the UVA irradiation conditions (see Materials and Methods). The major stop sites produced by the templates treated with **5** either in dark or under the irradiation are shown in Fig 5 (lanes **5-**dark and **5**-UV). These stop sites were similar to those produced by *cisplatin* (Fig 5, lane *cisplatin*), *i*.*e*. appeared mainly at GG or AG sites—the preferential DNA binding sites for this metallodrug [23]. The stop sites produced by *transplatin* (shown for comparative purposes) were less regular and appeared mainly at single G and C sites—the preferential DNA binding sites for this platinum complex [24]. Importantly, the efficiency to block the RNA polymerases differed significantly for the adducts formed by **5** in the dark and the adducts formed upon UVA irradiation. The adducts formed by **5** (at r_b_ = 0.003) under irradiation conditions were much more effective in inhibiting the RNA synthesis compared to the adducts formed by **5** in the dark at the same or even higher level of platination (r_b_ = 0.003 and 0.01) [cf. lanes **5**-UV (0.003), **5**-dark (0.003) and **5**-dark (0.01) in Fig 5. Similar results were obtained also for complex **3** (not shown).

#### Interstrand DNA Cross-links

**Fig 6:**
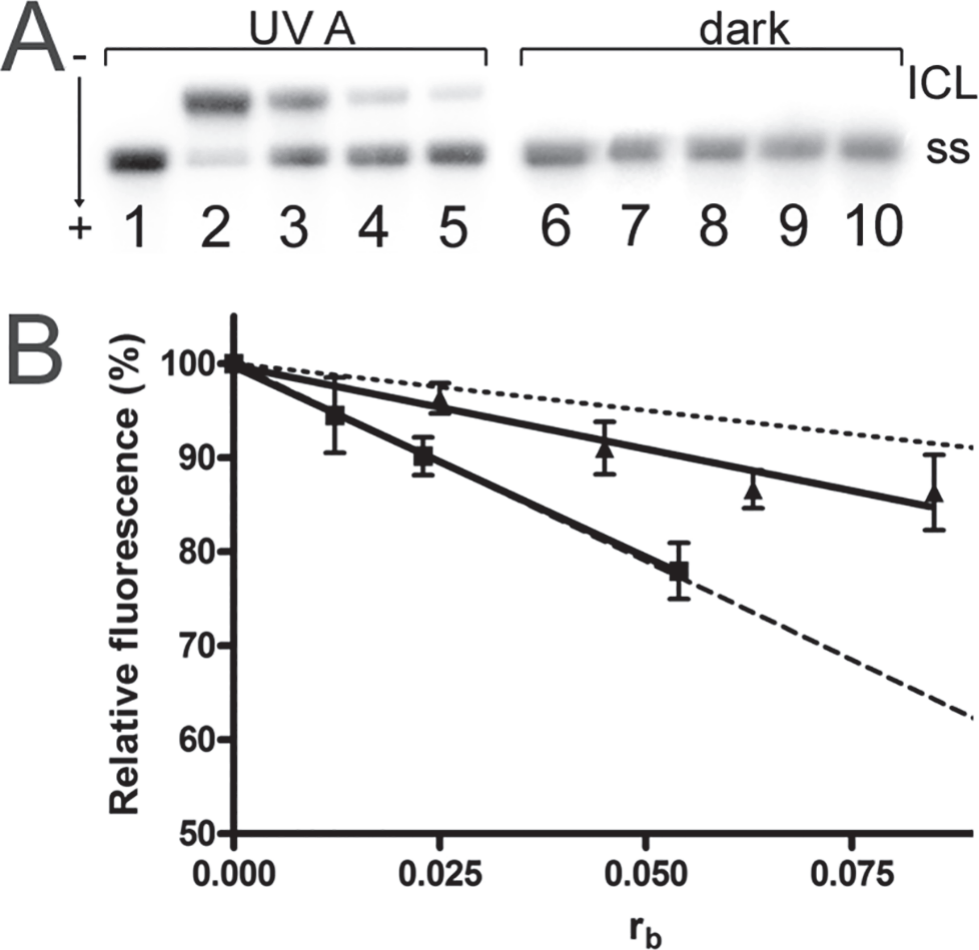
Formation of interstrand cross-links and dependence of ethidium bromide (EtBr) fluorescence. A. The formation of interstrand cross-links by complex **5** under the irradiation with UVA (lanes 1–5) or in the dark (lanes 6–10). Lanes: 1, control, untreated DNA (incubated under irradiation conditions); 2–5, r_b_ = 0.0027, 0.0013, 0.0007 and 0.0004, respectively; 6, control, untreated DNA (incubated in the dark); 7–10, r_b_ = 0.0033, 0.0016, 0.0009 and 0.0007, respectively. B. Dependence of ethidium bromide (EtBr) fluorescence on r_b_ for DNA modified by irradiated **5** (squares) or by **5** in the dark (triangles). Data are average ± SD for three independent experiments. Data for *cisplatin* (dashed line) and monofunctional *dienplatin* (dotted line) recorded under identical experimental conditions are taken from the literature [25].

Bifunctional platinum compounds, which coordinate to the base residues in DNA, form various types of interstrand and intrastrand cross-links. Such cross-links in the target DNA are important factors involved in the DNA damaging action of the genotoxic agents. Therefore, we have quantified the interstrand cross-linking efficiency of **3** or **5** when photoactivated or in the dark using linear pSP73KB/EcoRI DNA. The DNA samples were treated with complex **3** or **5** in dark or under the UVA irradiation conditions as described above. Samples were analyzed by agarose gel electrophoresis under denaturing conditions. The interstrand cross-linked DNA appears in the autoradiogram as the top bands (Fig 6A), as it migrates more slowly than the single-strand DNA (the bottom bands). The frequencies of interstrand cross-links formed by photoactivated **3** and **5** were 8±2, and 10±3%, respectively. Interestingly, the modification of DNA in dark resulted in the absence of the slowly migrating bands, indicating that these samples contained no detectable interstrand cross-linking, although the levels of platination (r_b_) were similar to those in the irradiated samples.

#### Characterization of DNA Adducts by Fluorescence Experiments

Ethidium bromide (EtBr), as a fluorescent probe, can be used to characterize the DNA binding of small molecules, such as platinum antitumor drugs and to distinguish the bifunctional from monofuntional DNA–adducts of platinum complexes [21,22]. Binding of EtBr to DNA by intercalation is blocked in a stoichiometric manner by the formation of bifunctional adducts of a series of platinum complexes, including *cisplatin*, which results in a loss of fluorescence intensity. However, DNA binding of monofunctional complexes such as *dienplatin* (chlorido-diethylenetriamineplatinum(II) chloride) results only in small decrease of the fluorescence intensity [25]. Modification of DNA by **3** or **5** under irradiation conditions resulted in a decrease of EtBr fluorescence (shown in Fig 6B for **5**) similar to that caused by *cisplatin* at equivalent r_b_ values. On the contrary, the decrease caused by the adducts of **5** formed in the dark was only slightly higher at equivalent r_b_ values than that induced by monodentately DNA-binding *dienplatin* (having only one leaving ligand). The analogous results were obtained also for the complex **3**.

## Discussion

The studied [Pt(cbdc)(*n*aza)_2_] complexes (**1**–**6**; Fig 1), prepared by Dhara´s method [17], represent the derivatives of the clinically used platinum-based drug *carboplatin* involving 7-azaindole (*n*aza) derivatives as *N*-donor carrier ligands. The coordination mode of the *n*aza ligands in the studied complexes was determined to be through the N7 atom as in the previous works reported in the X-ray structures of the platinum(II) dichlorido [11,26] and oxalato [26] complexes with the analogous *n*aza ligands. The same coordination mode was clearly proven also for the herein reported complexes **1**–**6** by the ^1^H and ^13^C NMR coordination shifts (S1 Table).

The prepared complexes showed high (against ovarian carcinoma cells) or moderate (against both the prostate carcinoma cancer cell lines) *in vitro* cytotoxicity (Table 1). In comparison to the recently studied dichlorido complexes (IC_50_ = 1.8–2.6 μM against A2780 and 1.5–3.8 μM against LNCaP cells [11]) with analogous *N*-donor ligands, the complexes studied in this work (**1**–**6**) are less effective against the mentioned human cancer cell lines.

One of the hot-topics in the field of platinum bioinorganic and medicinal chemistry is the preparation of agents having good stability and no or very low cytotoxic effect (so called prodrugs) and study their activation towards biologically active species [27]. Obviously, the studied complexes were not found to be inactive, but their cytotoxicity was still markedly lower as compared to their dichlorido analogues [11,13], which meant that there is still room to improve the biological activity of the studied *carboplatin*-analogues. To reach this goal, we used two of several possible strategies which can be applied to increase the biological activity of cytotoxic transition metal complexes, the first one was based on the addition of L-BSO (a selective inhibitor of γ-glutamylcysteine synthase), effectively blocking the inactivation of the complexes by GSH conjugation, and the second one using the photoactivation of the studied compounds by UVA light.

The complex **3** showed *ca*. 4.4-fold enhancement of *in vitro* cytotoxicity when it was administrated to the A2780 cells together with 5.0 μM L-BSO. As L-BSO is a well-known inhibitor of γ-glutamylcysteine synthase, it has a profound effect on the mechanism of action of the cytotoxic transition metal complexes having either *cisplatin*-like or redox processes modulating mechanism of action [18,28,29]. In other words, decreasing of the GSH cellular levels, caused by the L-BSO addition, affects the cytotoxicity of the complexes inactivated by a GSH-mediated cellular detoxification (*e*.*g*. *cisplatin* in the *cisplatin*-resistant cancer cell lines) as well as the cytotoxicity of the complexes, whose biological effect is mediated through the cellular redox processes. Since it has been proved by the ^1^H NMR and ESI-MS experiments that the studied complexes do not interact with GSH, the increase of the biological effect of **3** on the A2780 cancer cells could come from the modulation of other cellular redox pathways.

Iit is known for *carboplatin* that its *in vitro* cytotoxicity could be enhanced by the UVA irradiation [8]. That is why we decided to study the UVA irradiation effect on the biological profile of the herein studied *carboplatin* derivatives. We chose A2780 and LNCaP cells, which were exposed to **2**–**5** for 24 h, followed by 20 min irradiation with UVA or sham irradiation. We observed that upon UVA irradiation, the *in vitro* cytotoxicity of the tested substances increased markedly, as compared with the experiments performed in dark (Fig 3). These promising results motivated us to further perform more detailed molecular biological and biophysical studies to uncover the mechanistic aspects responsible for the considerable biological activity of the studied complexes.

The studied compounds were stable in DMF-*d*_*7*_ over 14 days, as judged by ^1^H NMR spectra. On the other hand, analogical experiments performed in the DMF-*d*_*7*_/H_2_O mixture (1:1, v/v) provided one new N1–H signal of the *4Br*aza *N*-donor ligand (11.94 ppm) after 24 h (Fig 2). The chemical shift value differs from that of free *4Br*aza (11.82 ppm), which suggested (together with the fact that only one new signal was detected—release of the *4Br*aza molecule from complex **5** would have to led to at least two new signals) that the mentioned N1–H signal belongs to the *4Br*aza ligand coordinated in the species having different composition from that of the starting material. Speculatively, the mentioned species could contain an open six-membered PtO_2_C_3_ ring (formed by the central atom and bidentate-coordinated cbdc ligand within the starting complex **5**) and could correspond to the composition *cis*-[Pt(*4Br*aza)_2_(cbdc`)(H_2_O)] (cbdc`= monodentate-coordinated cbdc ligand), which was both experimentally [30,31] and theoretically [32] proven for *carboplatin*, but we did not get any evidence for this process (*e*.*g*. from ESI-MS performed on this sample) in the case of herein reported *carboplatin* derivatives.

With an intention to better understand the composition and behavior of the studied complexes (represented by the selected complex **5**) before and after the UVA irradiation, as well as in the presence of GSH, GMP or genomic DNA, we decided to perform the stability and interaction studies (^1^H NMR, ESI-MS) of **5** and its mixtures with GSH (one of the major reducing sulfur-containing agents of human plasma with known coordination affinity towards Pt(II) atom, representing both the transport opportunities and some ways of inactivation of Pt(II) anticancer drugs) or GMP (well-known model system of the target binding site on DNA molecule attacked by the cytotoxic platinum(II) complexes). In the case where the studied complex was irradiated by UVA light (λ_max_ = 365 nm) for 20 min, two new N1–H signals of *4Br*aza were detected in the ^1^H NMR spectra at 12.35 and 11.82 ppm together with the signal of the starting material at 13.02 ppm, while the signal at 11.94 ppm (as detected for the unirradiated complex **5**) was not found (Fig 2). One of these signals (11.82 ppm) belongs to the free *4Br*aza molecule, as proved by the ^1^H NMR experiments with the irradiated solution of **5** spiked with the solution of free *4Br*aza ligand (Fig 4). This is also consistent with the results of ESI-MS, where the intensity of the peaks of the {[Pt(cbdc)(*4Br*aza)]–H}^—^and {*4Br*aza+H}^+^ fragments, whose formation is directly associated with a release of the *N*-donor ligand from the parent complex, markedly increased after the UVA irradiation (S5 Fig). The second new peak (12.35 ppm) split into two (12.35 and 12.18 ppm) after longer than 24 h standing at room temperature under ambient light (Fig 4). We believe that this change is caused by rearrangement or isomerization (*e*.*g*. *cis*-to-*trans* transformation) of the species formed after the irradiation, and not by the formation of the new species with different composition, because the overall integral intensity of these two signals is in the same ratio to the other two N1–H peaks at 13.02 and 11.82 ppm, as in the case of the spectrum recorded 24 h after the irradiation. Further, this observation indirectly proved the opening of the six-membered PtO_2_C_3_ chelate ring, without which the above mentioned isomerization would not be possible. Opening of this chelate ring was also indicated by the ^1^H NMR results, because the chemical shift of one new C13–H_2_ quintet has been detected in the ^1^H NMR spectrum of the irradiated sample at 2.08 ppm (S4 Fig) differing from both the complex **5** and free H_2_cbdc molecule. With respect to the described findings, it can be assumed that the composition of the platinum-containing species formed from complex **5** after the UVA irradiation corresponds to *cis*-[Pt(H_2_O)_2_(cbdc`)(*4Br*aza)] (N1–H signal of *4Br*aza at 12.35 ppm), which partially rearranges with time to *trans*-[Pt(H_2_O)_2_(cbdc`)(*4Br*aza)] (12.18 ppm). Unfortunately, no direct evidence for these statements from the mass spectra of the irradiated sample was found, probably due to decomposition of the mentioned aqua species connected with the Pt–OH_2_ bond cleavage under electrospray ionization conditions.

Photoreaction with GSH does not lead to the formation of adducts of GSH with complex **5** or fragments, as judged by the ^1^H NMR and ESI-MS experiments (S5 Fig). Although the changes in the NMR and mass spectra before and after the irradiation were detected, they correspond only to irradiation (as discussed above for the original complex) and not to interaction with this sulfur-containing biomolecule. The statement that GSH does not interact with complex **5** or with the species formed from complex **5** after UVA irradiation can be proved also by the fact that no new N1–H signals of *4Br*aza were observed in the ^1^H NMR spectra, as compared to the ^1^H NMR spectra of **5** alone (as discussed above). Further, the platinum-containing adducts with GSH were not found by the ESI-MS before and after irradiation (S5 Fig).

On the other hand, GMP showed the ability to interact with Pt(II) atom in the representative complex **5**, since the ^1^H NMR spectra of the irradiated **5**+GMP mixture contained one new signal of the C8–H hydrogen atom of GMP at 8.31 ppm, as well as the signals of free *4Br*aza released from the parent complex (S6 Fig). Although one could expect the coordination of GMP to the Pt(II) atom (as known for *carboplatin* [30]), it could not be unambiguously judged based on the proton NMR spectra, because no new set of signals of *4Br*aza, coordinated within the GMP-containing species, was detected. Still, even if we could think about the substitution of both the *4Br*aza molecules involved in the starting complex by two GMP molecules to be possible, this process would be firstly not very probable, and secondly it was directly disproved by ESI-MS results showing the peaks assignable to the {[Pt(cbdc)(*4Br*aza)(GMP)]–Na+2H}^+^, {[Pt(cbdc)(*4Br*aza)(GMP)]+H}^+^ and {[Pt(cbdc)(*4Br*aza)(GMP)]–2Na+H}^—^species (S5 Fig). It has to be noted, that these peaks were identified only in the spectra of the irradiated samples, contrary to the peaks at 1118.2, 1140.2 and 1094.0 *m/z* of the species whose mass corresponds to {[Pt(cbdc)(*4Br*aza)_2_(GMP)]–Na+2H}^+^, {[Pt(cbdc)(*4Br*aza)_2_(GMP)]+H}^+^ and {[Pt(cbdc)(*4Br*aza)(GMP)]–2Na+H}^–^, respectively, which were detected in the appropriate spectra even before the UVA irradiation of the studied **5**+GMP mixture, which indicates that these adducts formed only as a consequence of the electrospray ionization process.

Since DNA is the major pharmacological target of the antitumor platinum drugs [2,33,34] it was also of great interest to examine whether the enhanced cytotoxicity of the complexes correlates with DNA binding of the photoactivated derivatives, similarly as in the case of *carboplatin* [8]. The initial experiments were aimed to quantify the binding of **1**–**6** and *carboplatin* to mammalian DNA in cell-free media. The results proved that the amount of platinum bound to DNA was markedly enhanced due to the UVA irradiation (after 5 h) as compared to the samples incubated in dark (Table 2). The results of DNA binding of *carboplatin* in dark and under continuous irradiation with UVA are in good agreement with the previously published data [8], confirming that less than 5% of *carboplatin* is bound to DNA in the sample which was kept in the dark. This is in contrast to the increased level of DNA-platination in the sample irradiated with UVA, so that more than 50% of platinum from *carboplatin* was bound after 5 h (Table 2). Notably, under comparable conditions, the amount of molecules of *carboplatin* bound to DNA was lower than that of molecules of **1–6**. Recent works have shown that the transcription on DNA templates modified by bidentate adducts of platinum complexes can be prematurely terminated at the level or in the proximity of such adducts, while the monofunctional DNA adducts of platinum complexes were unable to terminate the RNA synthesis [19,35,36]. So, the considerably different efficacy of DNA adducts formed by **3** or **5** in dark and under the irradiation conditions (at the same level of DNA platination) to inhibit RNA polymerase is consistent with different frequency of mono- and bifunctional adduct formed by these complexes in dark and under the irradiation conditions. Thus, the results of transcription mapping experiments (Fig 5) support the hypothesis that under irradiation conditions, complexes **3** and **5** preferentially form the bifunctional adducts with DNA, capable of effective termination of RNA synthesis by RNA polymerases. On the other hand, in dark, the formation of less effective monofunctional adduct prevails.

The results of transcription mapping experiments are in good agreement with the characterization of DNA adducts formed by **3** or **5** in the dark and under irradiation conditions estimated by the EtBr fluorescence quenching (Fig 6B). These results show that **3** and **5** form in the dark the DNA adducts which resemble, from the viewpoint of their capability to inhibit EtBr fluorescence, those formed by monofunctional platinum complexes. Notably, the DNA adducts formed by **3** and **5** under irradiation conditions inhibited EtBr fluorescence to the same extent as bifunctional *cisplatin*. Hence, the fluorescent analysis is consistent with the idea and supports the postulate that the major DNA adducts formed by **3** or **5** in dark are mainly monofunctional lesions. In contrast, under comparable conditions (at the same level of DNA platination), but under the irradiation conditions, **3** and **5** form on DNA mainly bifunctional adducts similar to those formed by *cisplatin*.

The latter conclusion is also reinforced by the observation (Fig 6A) that **3** or **5** formed a significant amount of bifunctional interstrand cross-links under the irradiation of DNA (even slightly higher than *cisplatin* at the same level of platination [19]) whereas **3** or **5** formed under comparable conditions no such bifunctional lesions in the dark in DNA.

In addition, the results characterizing the monofunctional binding of **3** or **5** to highly polymeric double-helical DNA are consistent with the formation of a ring-opened species [Pt(cbdc`)(*n*aza)_2_(H_2_O)], containing monodentate cbdc`, in the dark. The bifunctional cross-links are probably formed as a consequence of the photoactivation by UVA and very likely occur as a consequence of the reaction of DNA with bifunctional *cis*-[Pt(H_2_O)_2_(cbdc`)(*n*aza)] active intermediate (containing two easily exchangeable H_2_O ligands). The structure of the such bifunctional product was suggested on the basis of ^1^H NMR and ESI-MS characterization of the UVA-irradiated solutions of complex **5** (*vide supra*).

To conclude the presented work, we prepared and characterized a series of *carboplatin* derivatives, involving the halogeno-substituted 7-azaindoles as the *N*-donor carrier ligands. The *in vitro* cytotoxicity of the prepared complexes against A2780 human ovarian carcinoma cell line was markedly (*ca*. 4.4-times) increased by the addition L-BSO. Based on the results of the detailed ^1^H NMR and ESI-MS studies carried out on the starting complex **5** and its mixtures with biomolecules GSH or GMP, as well as on the results of DNA-platination, it is obvious that UVA irradiation (20 min, λ_max_ = 365 nm) led to the release of one *4Br*aza ligand and hydrolysis of one of the Pt–O bonds between central Pt(II) atom and the chelating cbdc dianion. Additionally, the UVA irradiation led to subsequent formation of the activated species (most probably *cis*-[Pt(H_2_O)_2_(cbdc`)(*n*aza)]) and resulted in markedly higher cytotoxicity of **5** against A2780 ovarian carcinoma and LNCaP prostate adenocarcinoma human cancer cell lines, as compared with sham-irradiated samples. Moreover, DNA binding of the studied complexes is markedly enhanced by the irradiation, as was proven on both chemical (ability to interact with GMP) and biological (higher CT DNA platination) experimental levels. Thus, in connection with the acquired results we have reason to believe that the complexes **1**–**6** could represent suitable candidates for use in photoactivated cancer chemotherapy.

## Supporting Information

S1 Fig ^1^H NMR, ^13^C NMR, ^1^H–^1^H gs-COSY, ^1^H–^13^C gs-HMQC and ^1^H–^13^C gs-HMBC spectra of 5. The ^1^H-NMR (up left), ^13^C-NMR (up right), ^1^H–^1^H gs-COSY (middle left), ^1^H–^13^C gs-HMQC (middle right) and ^1^H–^13^C gs-HMBC (down) spectra obtained on the solution of **5** in DMF-*d*_*7*_; the chemical shift values are given in Experimental section in the main text.
(TIF)

S2 Fig ESI+ mass spectrum of 5. ESI+ mass spectrum (0–800 *m/z* range) of the methanolic solution of the complex **5** (A) and its part between 720 and 765 *m/z* showing the molecular peak (together with isotopic distribution) and its adduct with sodium ion observed experimentally (B) and calculated (C).
(TIF)

S3 Fig Time-dependent ^1^H NMR spectra before and after UVA irradiation of 5. Time-dependent (fresh solution and after 24 h) 400 MHz ^1^H NMR spectra as observed before and after UVA irradiation (20 min, 365 nm) of the complex **5** dissolved in the DMF-*d*_*7*_/H_2_O solution (1:1, *v/v*).
(TIF)

S4 Fig ESI+ and ESI—mass spectra of 5 and its mixtures with GSH or GMP with or without UVA irradiation. ESI+ (left) and ESI—(right) mass spectra (100–1200 *m/z* range) of the complex **5** and its mixtures with GSH or Na_2_GMP (dissolved in the DMF-*d*_*7*_/H_2_O, 1:1, *v/v*) as detected on the samples with or without irradiation (20 min, 365 nm) 24 h after preparation. ♦ stands for {*4Br*aza+H}^+^, × for {GMP–Na+2H}^+^, {GMP+H}^+^ or {GMP–2Na–H}^–^, ● for {GS–SG+H}^+^ or {GS–SG+Na}^+^, ○ for {[Pt(cbdc)(*4Br*aza)_2_]+H}^+^, {[Pt(cbdc)(*4Br*aza)_2_]+Na}^+^ or {[Pt(cbdc)(*4Br*aza)_2_]–H}^–^, ♯ for {[Pt(cbdc)(*4Br*aza)]–H}^–^, and ◊ for {[Pt(cbdc)(*4Br*aza)(GMP)]–Na+2H}^+^, {[Pt(cbdc)(*4Br*aza)(GMP)]+H}^+^ or {[Pt(cbdc)(*4Br*aza)(GMP)]–2Na–H}^–^.
(TIF)

S5 Fig ^1^H NMR spectrum after UVA irradiation of the mixture of 5 and GMP. 400 MHz ^1^H NMR spectrum as observed after UVA irradiation (20 min, 365 nm) of the mixture of the complex **5** and GMP dissolved in the DMF-*d*_*7*_/H_2_O solution (1:1, *v/v*).
(TIF)

S6 Fig ESI—mass spectrum of the {[Pt(cbdc)(*4Br*aza)(GMP)]–2Na+H}^—^species. Experimental (up) and simulated (down) mass spectrum isotope distribution of the {[Pt(cbdc)(*4Br*aza)(GMP)]–2Na+H}^—^species detected in the ESI—mass spectrum of the complex **5** and GMP mixture dissolved in the DMF-*d*_*7*_/H_2_O solution (1:1, *v/v*). The fresh mixture was irradiated (20 min, 365 nm) and the spectrum was recorded 24 h after preparation.
(TIF)

S7 Fig Impact of UVA irradiation of platinum(II) carboxylato complexes with 7-azaindoles as carrier ligands on their cytotoxicity. (TIF)

S1 Table The ^1^H and ^13^C NMR coordination shifts (calculated as Δδ = δ_complex_—δ_ligand_; ppm) of the prepared complexes The ^1^H and ^13^C NMR coordination shifts (calculated as **Δ**δ = δ_complex_—δ_ligand_; ppm) of the prepared complexes.
(PDF)

S1 Text The characterization data (^1^H and ^13^C NMR, elemental analysis, FTIR and ESI-MS) for 1–6. (PDF)
