# Supplementary figures and images for: Potentiating Effect of UVA Irradiation on Anticancer Activity of Carboplatin Derivatives Involving 7-Azaindoles
Source: PLoS One. 2015 Apr 15;10(4):e0123595. doi: 10.1371/journal.pone.0123595 (PMC4398499; doi:10.1371/journal.pone.0123595)

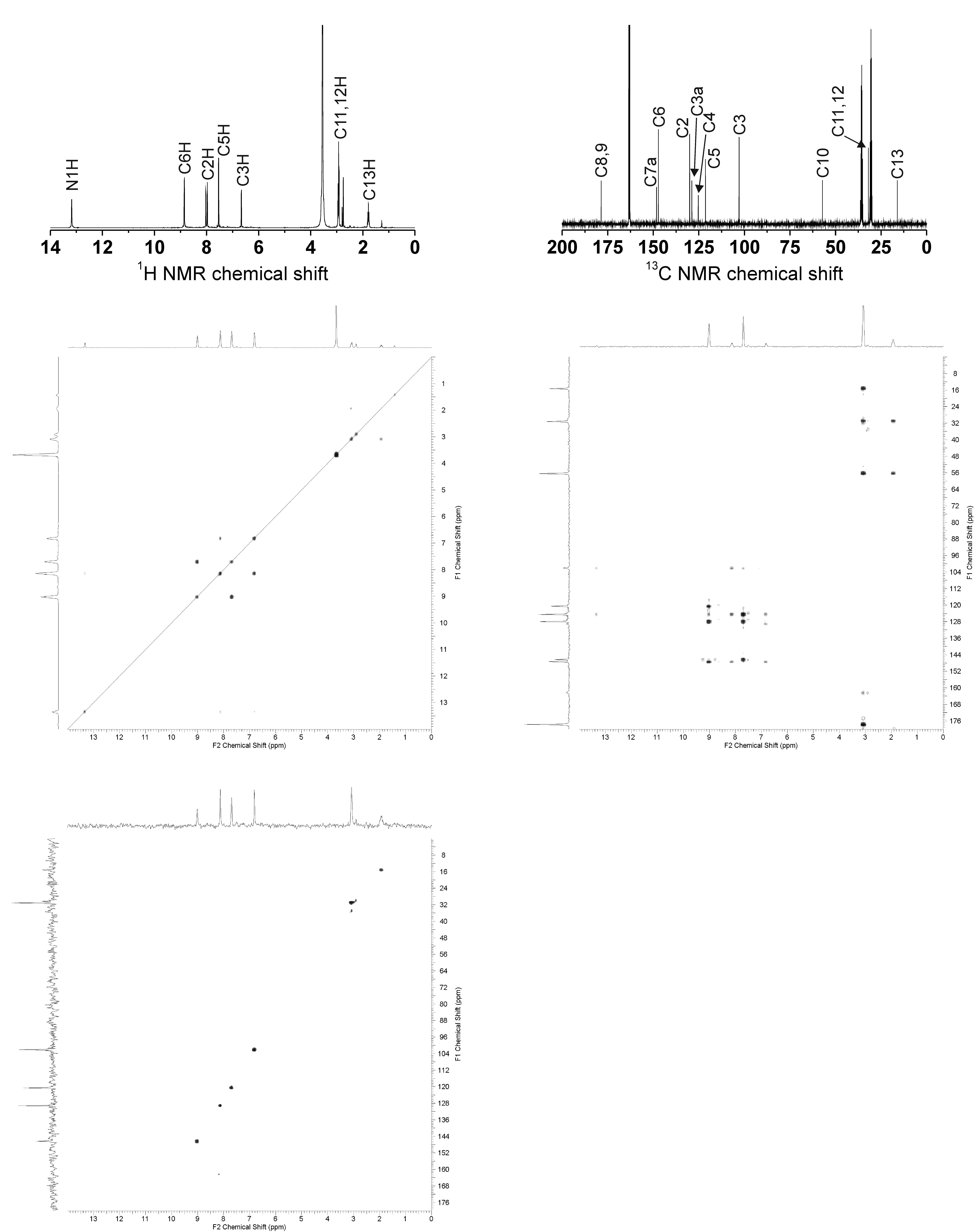

Supplement: S1 Fig — The 1H-NMR (up left), 13C-NMR (up right), 1H–1H gs-COSY (middle left), 1H–13C gs-HMQC (middle right) and 1H–13C gs-HMBC (down) spectra obtained on the solution of 5 in DMF-d 7; the chemical shift values are given in Experimental section in the main text. (TIF) [file pone.0123595.s001.tif]

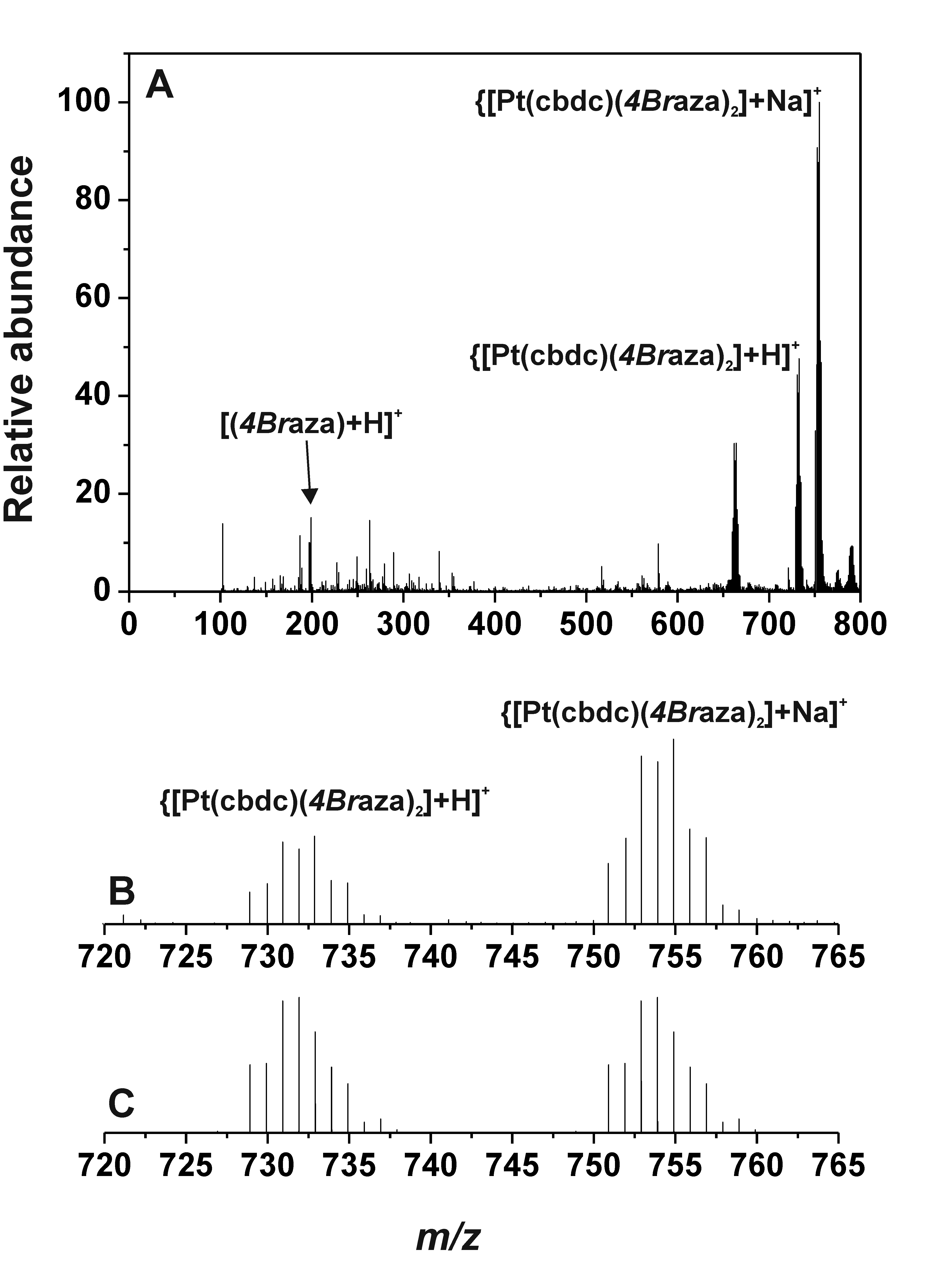

Supplement: S2 Fig — ESI+ mass spectrum (0–800 m/z range) of the methanolic solution of the complex 5 (A) and its part between 720 and 765 m/z showing the molecular peak (together with isotopic distribution) and its adduct with sodium ion observed experimentally (B) and calculated (C). (TIF) [file pone.0123595.s002.tif]

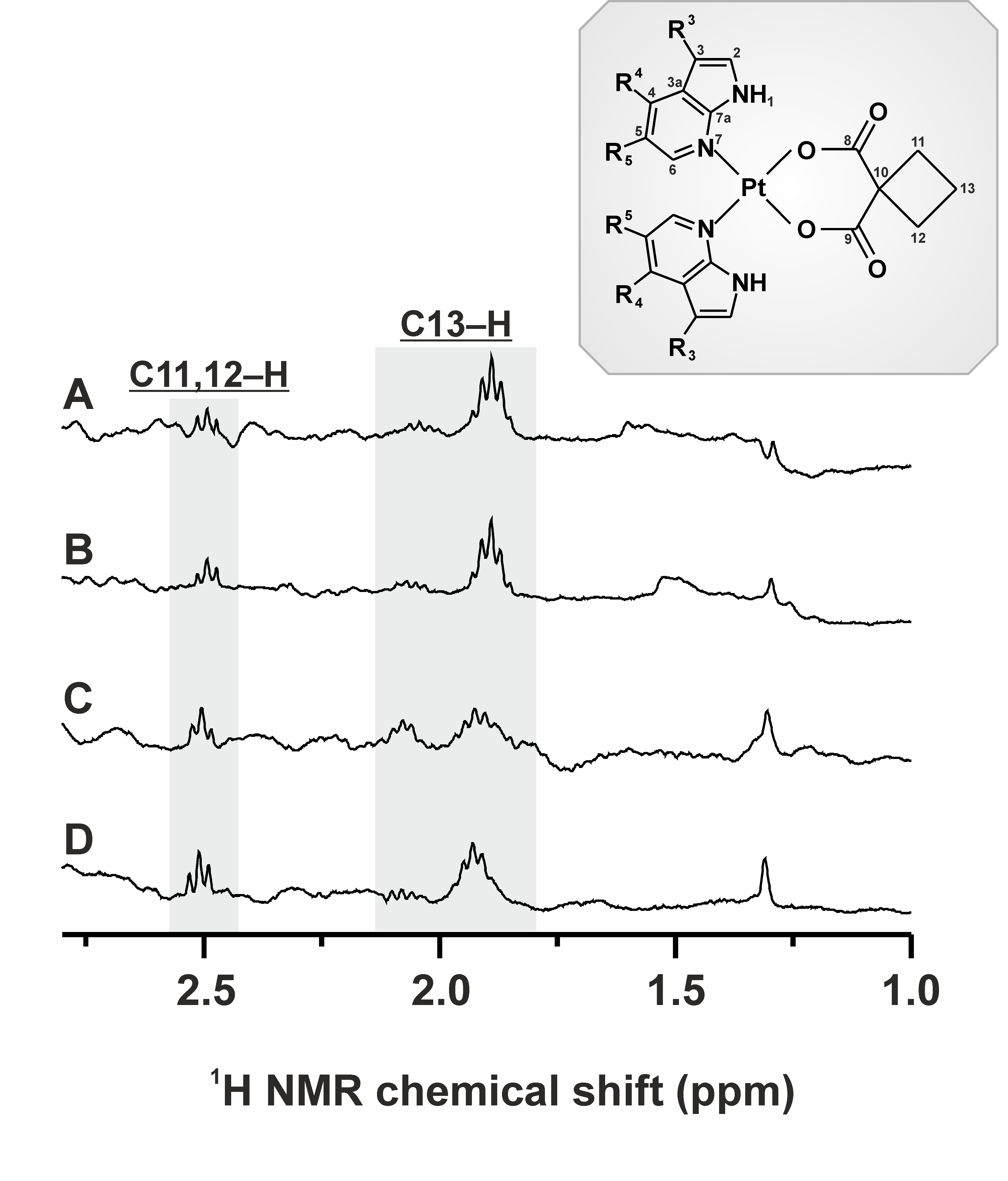

Supplement: S3 Fig — Time-dependent (fresh solution and after 24 h) 400 MHz 1H NMR spectra as observed before and after UVA irradiation (20 min, 365 nm) of the complex 5 dissolved in the DMF-d 7/H2O solution (1:1, v/v). (TIF) [file pone.0123595.s003.tif]

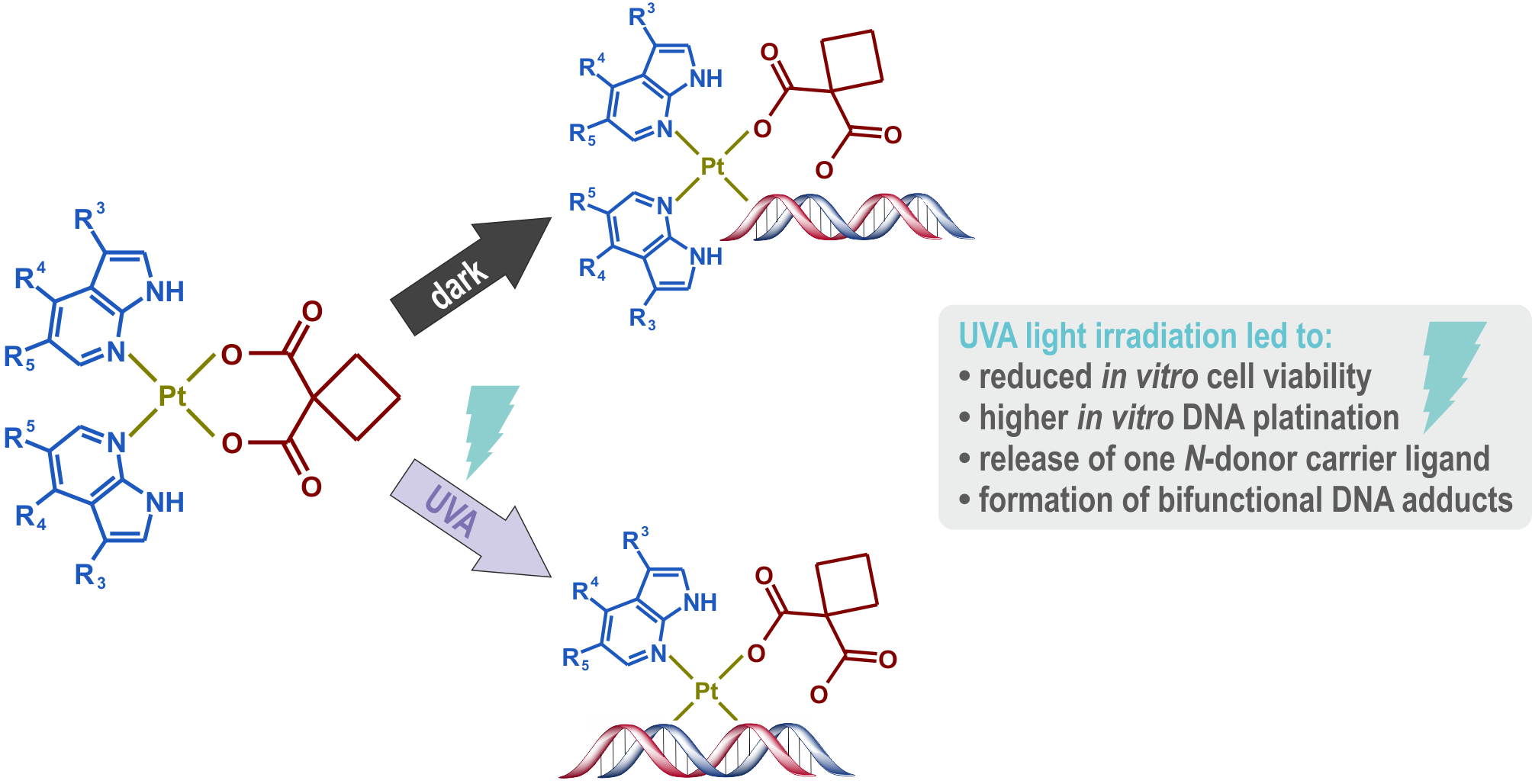

Supplement: S7 Fig — (TIF) [file pone.0123595.s007.tif]
