# Supplementary material for: Potentiating Effect of UVA Irradiation on Anticancer Activity of Carboplatin Derivatives Involving 7-Azaindoles
Source: PLoS One. 2015 Apr 15;10(4):e0123595. doi: 10.1371/journal.pone.0123595 (PMC4398499; doi:10.1371/journal.pone.0123595)
Supplement: S1 Table — The 1H and 13C NMR coordination shifts (calculated as Δδ = δcomplex—δligand; ppm) of the prepared complexes. (PDF) [file pone.0123595.s008.pdf]

	^{1}H NMR						^{13}C NMR						
	N1H	C2H	C3H	C4H	C5H	C6H	C2	C3	C3a	C4	C5	C6	C7a
**1**	1.45	0.32	–	0.20	0.16	0.72	2.7	2.7	4.0	4.8	2.2	4.1	–0.1
**2**	1.07	0.30	–	0.19	0.14	0.73	2.6	2.5	4.0	4.7	2.2	4.2	0.0
**3**	1.10	0.28	–	0.17	0.14	0.73	2.5	2.6	4.0	4.7	2.0	4.0	0.1
**4**	1.11	0.28	0.17	–	0.21	0.74	2.5	3.0	3.7	4.6	2.6	3.9	–0.8
**5**	1.09	0.27	0.16	–	0.19	0.73	2.6	3.0	4.9	3.9	2.6	3.8	–0.7
**6**	1.26	0.29	0.19	0.23	–	1.09	2.4	2.9	3.3	4.8	0.1	3.8	0.6
