# Supplementary material for: Potentiating Effect of UVA Irradiation on Anticancer Activity of Carboplatin Derivatives Involving 7-Azaindoles
Source: PLoS One. 2015 Apr 15;10(4):e0123595. doi: 10.1371/journal.pone.0123595 (PMC4398499; doi:10.1371/journal.pone.0123595)
Supplement: S1 Text — (PDF) [file pone.0123595.s009.pdf]

[Pt(cbdc)(*3Cl*aza)$_2$] (**1**): Grey powder; yield 38%. ^{1}H NMR (400.00 MHz, DMF-d_7, ppm): δ = 13.15 (br s, N1–H), 9.07 (d, *J* = 4.8 Hz, C6–H), 8.17 (d, *J* = 7.6 Hz, C4–H), 8.05 (s, C2–H), 7.36 (t, *J* = 6.2 Hz, C5–H), 2.95 (t, J = 7.8 Hz, C11–H$_2$, C12–H$_2$), 1.78 (qui, J = 7.8 Hz, C13–H$_2$). ^{13}C NMR (100.58 MHz, DMF-d_7, ppm): δ = 178.7 (C8, C9), 148.4 (C6–H), 147.3 (C7a), 130.6 (C4–H), 126.1 (C2–H), 121.7 (C3a), 118.6 (C5–H), 105.3 (C3), 57.11 (C10), 31.9 (C11–H$_2$, C12–H$_2$), 16.02 (C13–H$_2$). Elemental analysis calculated (%) for $C_{20}H_{16}N_4Cl_2O_4Pt$: C 37.40, H 2.51, N 8.72. Found: C 37.52, H 2.55, N 8.42. FTIR (ν_{ATR}/cm^{-1}): 442w, 477w, 520w, 560w, 600m, 662m, 697w, 750s, 793m, 896w, 953w, 1017s, 1100m, 1206s, 1278s, 1338vs, 1364s, 1444m, 1492w, 1594vs, 1630vs, 2946s, 3099s. ESI+ MS (methanol, *m/z*): 664.0 (calc. 664.0; 20%) [{Pt(cbdc)(*3Cl*aza)$_2$}+Na]$^+$, 642.1 (calc. 642.0; 10%) [{Pt(cbdc)(*3Cl*aza)$_2$}+H]$^+$; 153.1 (calc. 153.0; 5%) [(*3Cl*aza)+H]$^+$. ESI– MS (methanol, *m/z*): 676.0 (calc. 676.0; 5%) [{Pt(cbdc)(*3Cl*aza)}+Cl]$^-$; 640.0 (calc. 640.0; 100%) [{Pt(cbdc)(*3Cl*aza)}–H]$^-$; 488.1 (calc. 488.0; 10%) [{Pt(cbdc)(*3Cl*aza)}–H]$^-$.

[Pt(cbdc)(*3Br*aza)$_2$] (**2**): Grey powder; yield 37%. ^{1}H NMR (400.00 MHz, DMF-d_7, ppm): δ = 13.24 (br s, N1–H), 9.07 (d, *J* = 5.7 Hz, C6–H), 8.08 (s, C2–H), 8.07 (m, C4–H), 7.35 (t, *J* = 5.8 Hz, C5–H), 2.94 (t, *J* = 7.8 Hz, C11–H$_2$, C12–H$_2$), 1.78 (qui, *J* = 7.8 Hz, C13–H$_2$). ^{13}C NMR (100.58 MHz, DMF-d_7, ppm): δ = 178.6 (C8, C9), 148.4 (C6–H), 147.9 (C7a), 131.4 (C2–H), 128.6 (C4–H), 123.3 (C3a), 118.8 (C5–H), 90.2 (C3), 57.2 (C10), 32.0 (C11–H$_2$, C12–H$_2$), 16.1 (C13–H$_2$). Elemental analysis calculated (%) for $C_{20}H_{16}N_4Br_2O_4Pt$: C 32.85, H 2.21, N 7.66. Found: C 32.51, H 1.96, N 7.20. FTIR (ν_{ATR}/cm^{-1}): 476w, 509w, 552w, 596m, 647m, 678w, 749s, 791m, 896w, 952w, 994s, 1055w, 1100m, 1154w, 1203m, 1274vs, 1334vs, 1441m, 1491m, 1592vs, 1632vs, 2948s, 3109s. ESI+ MS (methanol, *m/z*): 753.9 (calc. 753.9; 10%) [{Pt(cbdc)(*3Br*aza)$_2$}+Na]$^+$, 731.9 (calc. 731.9; 60%) [{Pt(cbdc)(*3Br*aza)$_2$}+H]$^+$; 197.0 (calc. 197.0; 20%) [(*3Br*aza)+H]$^+$. ESI– MS (methanol, *m/z*): 765.8 (calc. 765.9; 10%)

[{Pt(cbdc)(*3Br*aza)$_2$}+Cl]$^-$; 730.1 (calc. 729.9; 100%) [{Pt(cbdc)(*3Br*aza)}–H]$^-$; 532.1 (calc. 532.0; 15%) [{Pt(cbdc)(*3Br*aza)}–H]$^-$.

[Pt(cbdc)(*3I*aza)$_2$] (**3**): Light grey powder; yield 42%. ^{1}H NMR (400.00 MHz, DMF-*d$_7$*, ppm): δ = 13.28 (br s, N1–H), 9.04 (d, *J* = 5.7 Hz, C6–H), 8.09 (s, C2–H), 7.91 (d, *J* = 7.9 Hz, C4–H), 7.34 (dd, *J* = 7.9, 5.7 Hz, C5–H), 2.93 (t, *J* = 7.8 Hz, C11–H$_2$, C12–H$_2$), 1.78 (qui, *J* = 7.8 Hz, C13–H$_2$). ^{13}C NMR (100.58 MHz, DMF-*d$_7$*, ppm): δ = 178.6 (C8, C9), 148.6 (C7a), 148.2 (C6–H), 133.5 (C2–H), 133.0 (C4–H), 126.5 (C3a), 118.8 (C5–H), 90.2 (C3), 57.2 (C10), 32.0 (C11–H$_2$, C12–H$_2$), 16.1 (C13–H$_2$). Elemental analysis calculated (%) for $C_{20}H_{16}N_4I_2O_4Pt$: C 29.11, H 1.95, N 6.79. Found: C 29.04, H 2.07, N 7.17. FTIR (ν_{ATR}/cm^{-1}): 476w, 503w, 543w, 562w, 596w, 643w, 662w, 750m, 794m, 899w, 981m, 1057w, 1101m, 1155w, 1204m, 1272s, 1337vs, 1437m, 1490w, 1594vs, 1648vs, 2945m, 3124s. ESI+ MS (methanol, *m/z*): 847.8 (calc. 847.9; 60%) [{Pt(cbdc)(*3I*aza)$_2$}+Na]$^+$, 825.8 (calc. 825.9; 70%) [{Pt(cbdc)(*3I*aza)$_2$}+H]$^+$; 245.0 (calc. 245.0; 5%) [(*3I*aza)+H]$^+$. ESI– MS (methanol, *m/z*): 859.7 (calc. 859.9; 5%) [{Pt(cbdc)(*3I*aza)}+Cl]$^-$; 824.0 (calc. 823.9; 100%) [{Pt(cbdc)(*3I*aza)}–H]$^-$; 580.0 (calc. 579.9; 10%) [{Pt(cbdc)(*3I*aza)}–H]$^-$.

[Pt(cbdc)(*4Cl*aza)$_2$] (**4**): Grey powder; yield 46%. ^{1}H NMR (400.00 MHz, DMF-*d$_7$*, ppm): δ = 13.18 (br s, N1–H), 8.96 (d, *J* = 6.2 Hz, C6–H), 7.96 (t, *J* = 3.4 hz, C2–H), 7.41 (d, *J* = 6.2 Hz, C5–H), 6.75 (d, *J* = 3.4 Hz, C3–H), 2.93 (m, C11–H$_2$, C12–H$_2$), 1.78 (qui, *J* = 7.8 Hz, C13–H$_2$). ^{13}C NMR (100.58 MHz, DMF-*d$_7$*, ppm): δ = 178.6 (C8, C9), 149.0 (C7a), 147.4 (C6–H), 139.2 (C4), 129.9 (C2–H), 122.9 (C3a), 118.2 (C5–H), 101.3 (C3–H), 57.1 (C10), 31.9 (C11–H$_2$, C12–H$_2$), 16.0 (C13–H$_2$). Elemental analysis calculated (%) for $C_{20}H_{16}N_4Cl_2O_4Pt$: C 37.40, H 2.51, N 8.72. Found: C 37.03, H 2.16, N 8.75. FTIR (ν_{ATR}/cm^{-1}): 439w, 476m, 510w, 547w, 607m, 661w, 722s, 780m, 860s, 881w, 922w, 952m, 984w, 1071m, 1112m, 1169m, 1196s, 1227w, 1259s, 1326vs, 1340vs, 1371s, 1434m, 1468m, 1486m, 1505w, 1585vs, 1638vs, 2959s, 2989s,

3094s. ESI+ MS (methanol, *m/z*): 664.0 (calc. 664.0; 70%) [{Pt(cbdc)(*4Cl*aza)$_2$}+Na]$^+$, 642.0 (calc. 642.0; 35%) [{Pt(cbdc)(*4Cl*aza)$_2$}+H]$^+$; 153.1 (calc. 153.0; 5%) [(*4Cl*aza)+H]$^+$. ESI– MS (methanol, *m/z*): 640.2 (calc. 640.0; 100%) [{Pt(cbdc)(*4Cl*aza)}–H]$^-$; 488.2 (calc. 488.0; 25%) [{Pt(cbdc)(*4Cl*aza)}–H]$^-$.

[Pt(cbdc)(*4Br*aza)$_2$] (**5**): Light grey powder; yield 41%. ^{1}H NMR (400.00 MHz, DMF-*d*$_7$, ppm): δ = 13.18 (br s, N1–H), 8.86 (d, *J* = 6.2 Hz, C6–H), 7.97 (s, C2–H), 7.54 (d, *J* = 6.2 Hz, C5–H), 6.67 (s, C3–H), 2.95 (t, *J* = 7.9 Hz, C11–H$_2$, C12–H$_2$), 1.78 (qui, *J* = 7.9 Hz, C13–H$_2$). ^{13}C NMR (100.58 MHz, DMF-*d*$_7$, ppm): δ = 178.6 (C8, C9), 148.2 (C7a), 147.2 (C6–H), 130.0 (C2–H), 128.8 (C3a), 125.3 (C4), 121.4 (C5–H), 102.9 (C3–H), 57.2 (C10), 32.0 (C11–H$_2$, C12–H$_2$), 16.1 (C13–H$_2$). Elemental analysis calculated (%) for $C_{20}H_{16}N_4Br_2O_4Pt$: C 32.85, H 2.21, N 7.66. Found: C 31.99, H 2.02, N 7.30. FTIR (ν_{ATR}/cm^{-1}): 476w, 503w, 538w, 559w, 602w, 646w, 723s, 781m, 806m, 854s, 919w, 975w, 1050w, 1072w, 1113m, 1188s, 1219w, 1259m, 1323vs, 1340vs, 1373s, 1431w, 1482m, 1505w, 1584vs, 1640vs, 2959m, 2984m, 3092m, 3136m. ESI+ MS (methanol, *m/z*): 753.9 (calc. 753.9; 70%) [{Pt(cbdc)(*4Br*aza)$_2$}+Na]$^+$, 731.9 (calc. 731.9; 40%) [{Pt(cbdc)(*4Br*aza)$_2$}+H]$^+$; 197.0 (calc. 197.0; 10%) [(*4Br*aza)+H]$^+$. ESI– MS (methanol, *m/z*): 765.8 (calc. 765.9; 10%) [{Pt(cbdc)(*4Br*aza)$_2$}+Cl]$^-$; 730.1 (calc. 729.9; 100%) [{Pt(cbdc)(*4Br*aza)$_2$}–H]$^-$; 532.2 (calc. 532.0; 20%) [{Pt(cbdc)(*4Br*aza)}–H]$^-$.

[Pt(cbdc)(*5Br*aza)$_2$] (**6**): Dark grey powder; yield 48%. ^{1}H NMR (400.00 MHz, DMF-*d*$_7$, ppm): δ = 13.17 (br s, N1–H), 9.39 (s, C6–H), 8.43 (s, C4–H), 7.92 (s, C2–H), 6.69 (s, C3–H), 2.95 (t, *J* = 7.1 Hz, C11–H$_2$, C12–H$_2$), 1.78 (qui, *J* = 7.1 Hz, C13–H$_2$). ^{13}C NMR (100.58 MHz, DMF-*d*$_7$, ppm): δ = 178.6 (C8, C9), 148.1 (C7a), 146.7 (C6–H), 135.1 (C4–H), 130.6 (C2–H), 125.4 (C3a), 111.0 (C5), 102.9 (C3–H), 57.1 (C10), 32.0 (C11–H$_2$, C12–H$_2$), 16.0 (C13–H$_2$). Elemental analysis calculated (%) for $C_{20}H_{16}N_4Br_2O_4Pt$: C 32.85, H 2.21, N 7.66. Found: C 32.98, H 2.12, N 7.38. FTIR (ν_{ATR}/cm^{-1}): 470w, 495w, 515w, 549w, 601w, 704m, 728m, 780m, 884m, 908m,

947m, 1026w, 1067m, 1105m, 1167m, 1201m, 1269s, 1295s, 1341vs, 1473vs, 1504w, 1586vs, 1625vs, 2928vs, 2950vs, 3094s. ESI+ MS (methanol, *m/z*): 753.9 (calc. 753.9; 15%) $[\{Pt(cbdc)(5Braza)_2\}+Na]^+$, 732.0 (calc. 731.9; 10%) $[\{Pt(cbdc)(5Braza)_2\}+H]^+$; 197.0 (calc. 197.0; 5%) $[(5Braza)+H]^+$. ESI– MS (methanol, *m/z*): 765.9 (calc. 765.9; 10%) $[\{Pt(cbdc)(5Braza)_2\}+Cl]^-$; 730.0 (calc. 729.9; 100%) $[\{Pt(cbdc)(5Braza)_2\}-H]^-$; 532.1 (calc. 532.0; 25%) $[\{Pt(cbdc)(5Braza)\}-H]^-$.
